# Conjugate Addition of Grignard Reagents to Thiochromones Catalyzed by Copper Salts: A Unified Approach to Both 2-Alkylthiochroman-4-One and Thioflavanone

**DOI:** 10.3390/molecules25092128

**Published:** 2020-05-01

**Authors:** Tania J. Bellinger, Teavian Harvin, Ti’Bran Pickens-Flynn, Nataleigh Austin, Samuel H. Whitaker, Mai Ling C. Tang Yuk Tutein, Dabria T. Hukins, Nichele Deese, Fenghai Guo

**Affiliations:** 1 Department of Chemistry, Winston Salem State University, 601 S. Martin Luther King Jr. Dr., Winston Salem, NC 27110, USA; tbellinger115@rams.wssu.edu (T.J.B.); tharvin117@rams.wssu.edu (T.H.); tpickensflynn117@rams.wssu.edu (T.P.-F.); naustin116@rams.wssu.edu (N.A.); swhitaker118@rams.wssu.edu (S.H.W.); mtangyuktutein118@rams.wssu.edu (M.L.C.T.Y.T.); dhukins116@rams.wssu.edu (D.T.H.); ndeese119@rams.wssu.edu (N.D.); 2 Biomedical Research Infrastructure Center, Winston Salem State University, Winston Salem, NC 27110, USA

**Keywords:** conjugate addition, thiochroman-4-ones, Grignard reagents, thiochromones, thioflavanones, 2-alkylthiochroman-4-ones

## Abstract

Grignard reagents undergo conjugate addition to thiochromones catalyzed by copper salts to afford 2-substituted-thiochroman-4-ones, both 2-alkylthiochroman-4-ones and thioflavanones (2-arylthiochroman-4-ones), in good yields with trimethylsilyl chloride (TMSCl) as an additive. The best yields of 1,4-adducts can be attained with CuCN∙2LiCl as the copper source. Excellent yields of 2-alkyl-substituted thiochroman-4-ones and thioflavanones (2-aryl substituted) are attained with a broad range of Grignard reagents. This approach works well with both alkyl and aromatic Grignard reagents, thus providing a unified synthetic approach to privileged 2-substituted thiochroman-4-ones and a potential valuable precursor for further synthetic applications towards many pharmaceutically active molecules. The use of commercially available and/or readily prepared Grignard reagents will expedite the synthesis of a large library of both 2-alkyl substituted thiochroman-4-ones and thioflavanones for additional synthetic applications.

## 1. Introduction

The 1,4-conjugate addition reaction of organometallic reagents, including Grignard reagents, is one of the most reliable carbon–carbon bond formation reaction in organic synthesis. [1] Without the addition of catalysts, such as Cu (I) salts, Grignard reagents usually undergo 1,2-addition to α, β-unsaturated carbonyl compounds. It has been shown that with the addition of Cu(I) salts, Grignard reagents undergo exclusive 1,4-conjugate addition to carbonyl compounds to afford 1,4-adducts [1,2]. In this investigation, we aimed to develop a unified approach to a broad scope of 2-substituted thiochroman-4-ones by taking advantage of the ease in preparation and the breadth in the scope of Grignard reagents. Sulfur-containing heterocycles are widely present in numerous pharmaceutical active molecules as well as in many bioactive natural products [3,4,5,6], with widespread applications in areas, such as material science, biology, medicinal chemistry, and food chemistry, in recent years [7,8,9,10,11,12,13]. Although the isosteric replacement of an oxygen atom by a sulfur atom is expected to improve the bioavailability and bioactivity [14], sulfur-containing heterocyclic compounds are a much less studied area compared to oxygen-containing heterocycles. Due to their rich biological activities and widespread applications, the development towards an efficient synthesis of sulfur-containing compounds has gradually gained interest. In recent years, sulfur-containing heterocycles have been found to display rich biological activities, such as cytotoxic effects on tumor cells in vitro [15], the in vitro antileishmanial and cytotoxic activities [16], as well as the ability to kill tumor cells by inducing tumor cell apoptosis. [17] Thioflavonoids, the sulfur analogues of flavonoids [18,19,20,21], display many biological activities, such as antimicrobial, antioxidant, inhibition of nitric oxide production, and antifungal properties, etc. [22,23,24,25,26,27,28,29,30]. Many thiochromanone derivatives have been known to be effective bioreductive alkylating agents [25]. Thiochromanones, i.e., thiochroman-4-ones, 2-alkylthiochroman-4-ones, and thioflavanone (2-arylthiochroman-4-ones), have become increasingly valuable synthons and vital precursors in organic synthesis for bioactive thiochromanone derivatives [31,32,33,34,35,36,37,38].

**Figure 1:**
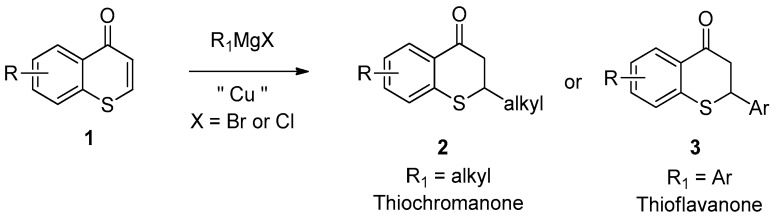
Copper-catalyzed conjugate addition of Grignard reagents to thiochromones.

Although the synthesis of sulfur-containing heterocycles is much less explored than oxygen-containing counterparts, increasingly more synthetic approaches to thiochroman-4-ones, thioflavone, thiochromones [39,40,41,42,43,44,45,46,47,48,49,50], and 2-substituted thiochroman-4-ones have been reported in recent years. The enantioselective synthesis of 2-arylsubstituted thiochromanone via Rh-catalyzed conjugate addition to thiochromones [51] and 2-alkylthiochromanones via the Cu-phosphoamidite-promoted enantioselective conjugate addition pathway has been reported recently [52]. A recent synthetic approach of thioflavanones by regioselective cyclization of 1-(2-benzylthio)phenyl-3-phenyl-2-propen-1-ones has also been published [53]. Other synthetic approaches to 2-substituted thiochroman-4-ones include the hydrogenation of thiochromones [54,55,56], intramolecular thio-Michael addition [57,58,59,60,61,62,63], and Friedel–Crafts acylation of thiopropanoic acid [64]. The synthesis of thiochroman-4-ones and thioflavanones via rhodium-catalyzed alkyne hydroacylation/thio conjugate addition sequence has also been reported [65]. With our interests in heterocycles, including sulfur-heterocycles, we also reported the synthesis of 2-alkylthiochromanones via conjugate addition of lithium dialkylcuprates to thiochromones [66] and a rapid entry to thioflavanones via the conjugate addition of diarylcuprates to thiochromones [67]. While most of these approaches provided efficient approaches to either thioflavanones (2-arylthiochroman-4-ones) or 2-alkylthiochromanone, there is a need for a unified approach that works well in introducing both the aliphatic and aromatic groups to furnish the desired 2-alkylthiochroman-4-ones **2** and thioflavanones (2-arylthiochroman-4-ones **3**). In pursuit of a unified approach of a broad scope of 2-substituted thiochroman-4-ones (**2**, R_1_ = alkyl and **3**, R_1_ = Ar; Figure 1) by taking advantage of the readily available and/or readily prepared Grignard reagents, both alkyl and aryl Grignard reagents, we now report the conjugate addition of Grignard reagents to thiochromones **1** to afford 2-substituted thiochroman-4-ones **2** and **3** in good yields using copper salts (Figure 1). This approach will allow access to a broader scope of thiochromanones and thioflavanones due to the readily available Grignard reagents compared to organolithium reagents.

## 2. Results and Discussions

**Scheme 1:**
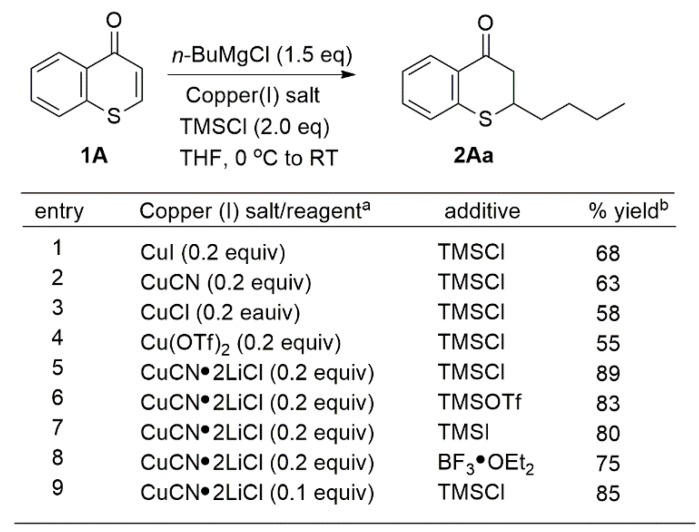
Optimization of 1,4-conjugate addition of *n*-butyl magnesium chloride to thiochromone. a. Reagents were prepared by adding *n*-BuMgCl to copper salts in THF. b. Yields are based on isolated products by column chromatography.

Our investigation started with *n*-BuMgCl to find the optimal reactional condition for 1,4-conjugate addition to thiochromone with copper salts. Our previous report showed that a Lewis acid activator, such as TMSCl, was needed in the conjugate addition of organolithium reagents to thiochromones using a stoichiometric amount of copper salts as no desired 1,4-adducts were observed in the absence of a Lewis acid activator [66,67]. A modest yield of 1,4-adduct 2-*n*-butylthiochroman-4-one **2Aa** was formed with *n*-BuMgCl and a 0.2 equivalent of CuI or CuCN with TMSCl as the activator (Scheme 1, entries 1–2). The use of CuCl did not improve the yield (Scheme 1, entry 3). With the addition of LiCl to CuCN, an excellent yield of 2-*n*-butylthiochroman-4-one **2Aa** was achieved in the presence of TMSCl (Scheme 1, entry 5, 89%). Other Lewis acids, such as TMSOTf, TMSI, and BF_3_∙OEt_2_, also activated this reaction but were not better activators than TMSCl as they did not offer better yields (Scheme 1, entries 6–8). These results show that the use of 0.2 equivalent of CuCN∙2LiCl and TMSCl offered the highest yield. We also found that CuCN∙2LiCl can be further reduced to 0.1 equivalent and a comparable yield of **2Aa** could be attained (Scheme 1, entry 9, 85%).

**Scheme 2:**
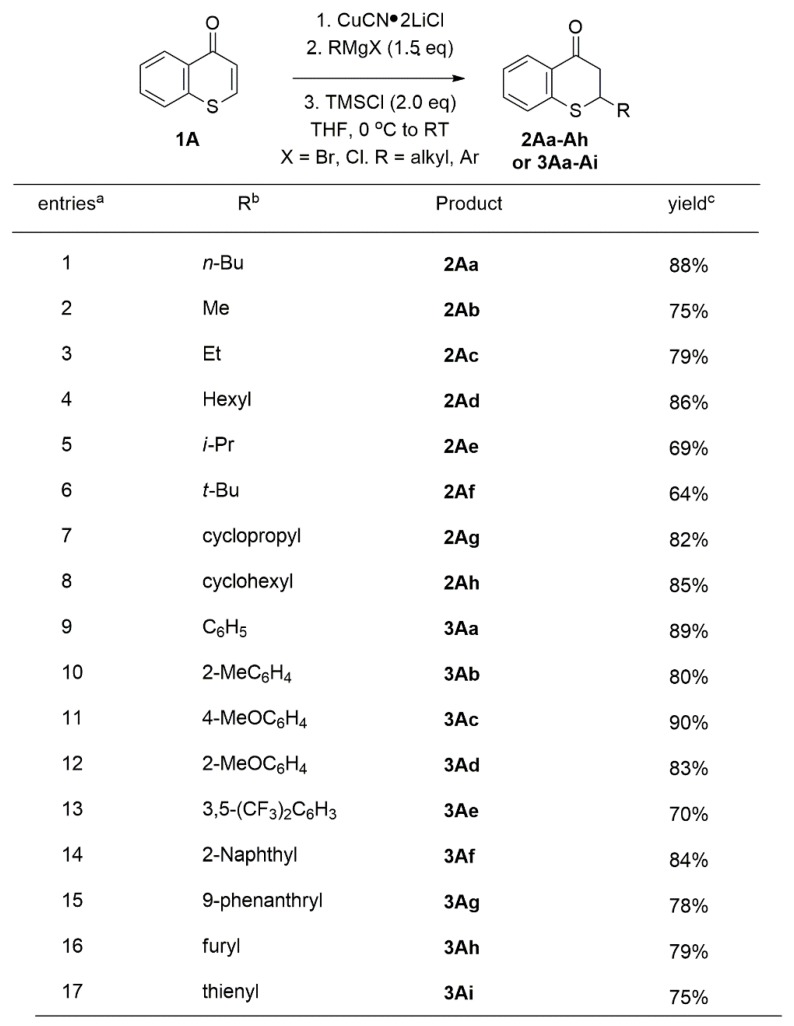
The scope of Grignard reagents in conjugate addition to thiochromone. a. All the reactions were performed using 1.5 equiv of RMgX in the presence of 2.0 equiv of TMSCI unless noted otherwise. b. RMgX were commercially available or prepared from corresponding RX in THF and used as a THF solution. c. Yields are based on isolated products by column chromatography.

With the optimal reaction condition in hand, the scope of the Grignard reagents was explored (Scheme 2, 64–90%). It was found that a broad range of Grignard reagents underwent conjugate addition to thiochromone **1A** to afford 1,4-adducts **2Aa**–**Ah** and **3Aa**–**Ai** with good reaction yields (Scheme 2). Simple Grignard reagents, such as methyl, ethyl, *n*-butyl, and *n*-hexyl Grignard reagents, were added to **1A** smoothly to afford 1,4-adducts with excellent efficiency (Scheme 2, 75–88%). Steric bulkier Grignard reagents, such as *i*-Pr, *t*-butyl Grignard reagents, gave lower yields (Scheme 2, **2Ae**, 69% and **2Af**, 64%). Cyclic Grignard reagents were also tolerated to give good yields of 1,4-adducts (Scheme 2, **2Ag**, 82% and **2Ah**, 85%). Aromatic Grignard reagents also reacted very well under these optimal reaction conditions. PhMgBr was added to thiochromone **1A** to offer 1,4-adduct 3Aa in an 89% yield. Grignard reagents with electron-donating groups on the aromatic ring were also found to work well (Scheme 2, **3Ab**–**Ad**, 80–90%). Grignard reagents with strong electron-withdrawing groups on the aromatic ring were also tolerated (Scheme 2, **3Ae**, 70%). Grignard reagents prepared from extended aryl bromides, such as 2-bromonaphthalene and 9-bromophenanthrene, were also added to thiochromone **1A** smoothly with high yields (Scheme 2, **3Af,** 84%; **3Ag**, 78%). To our delight, Grignard reagents prepared from aromatic heterocycles also worked well. Both (2-furyl)magnesium bromide and 2-thienylmagnesium bromide were added to thiochromone **1A** to afford corresponding 1,4-adducts in good yields (Scheme 2, **3Ah**, 79%; **3Ai**, 75%).

**Scheme 3:**
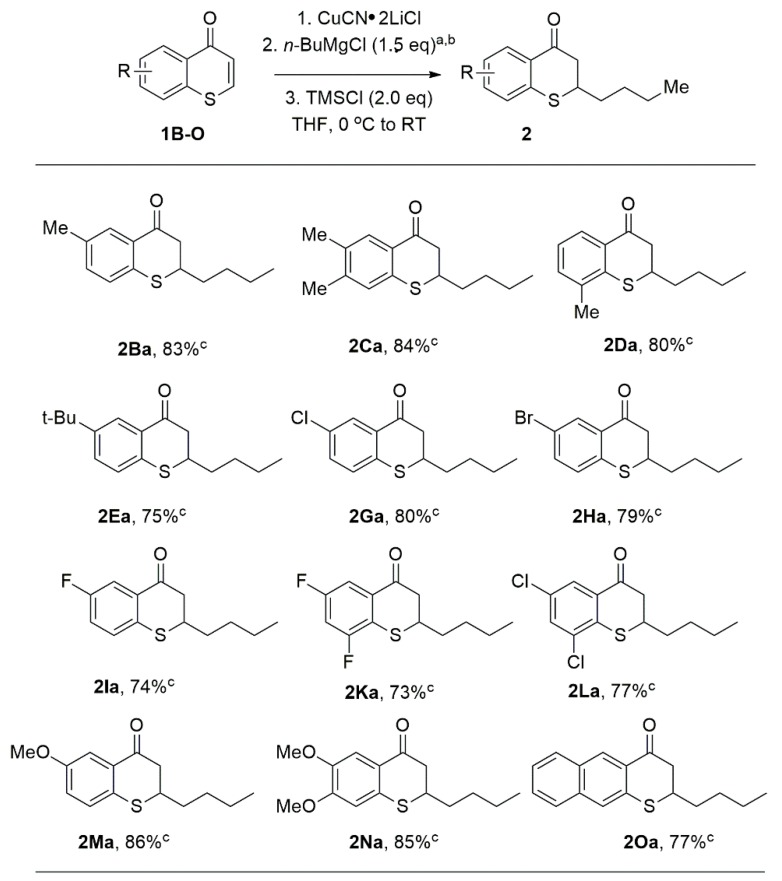
Scope of the substituted thiochromones with *n*-BuMgCl. a. All the reactions were performed using 1.5 equiv of BuMgCl in the presence of 2.0 equiv of TMSCI unless noted otherwise. b. RMgX were commercially available or prepared from corresponding RX in THF and used as a THF solution. c. Yields are based on isolated products by column chromatography.

With the optimal reaction addition for conjugate addition to thiochromone **1A** in hand, we next investigated the scope of thiochromone substrates for the conjugate addition of Grignard reagents. A number of substituted thiochromones **1B**–**1O** were investigated. It was found that *n*-butylmagnesium chloride readily added to the substituted thiochromones **1B**–**1O** to afford 1,4-adducts **2Ba**–**2Oa** with good yields (Scheme 3, 73–86%). Thiochromones bearing simple alkyl groups, such as a methyl group, reacted with *n*-BuMgCl to afford **2Ba**–**2Da** in good yields under the optimal reaction condition (Scheme 3, 80–84%). The bulky *t*-butyl group was also tolerated to afford 1,4-adduct **2Ea** with good yield. Thiochromones with halides, such as F, Br, and Cl, also reacted smoothly with *n*-BuMgCl (Scheme 3, 74–80%). Thiochromones with two halides on the aromatic ring, such as 6,8-difluorothiochromanone and 6,8-dichlorothiochromone, were also tolerated (Scheme 3, **2Ka**, 73% and **2La**, 77%). Electron-donating groups, such as MeO-, also worked well to afford 1,4-adduct **2Ma** and **2Na** in higher yields (Scheme 3). Thiochromane **1O** with an extended aromatic structure also underwent conjugate addition with *n*-BuMgCl to afford 1,4-adduct **2Oa** in a 77% yield (Scheme 3).

**Scheme 4:**
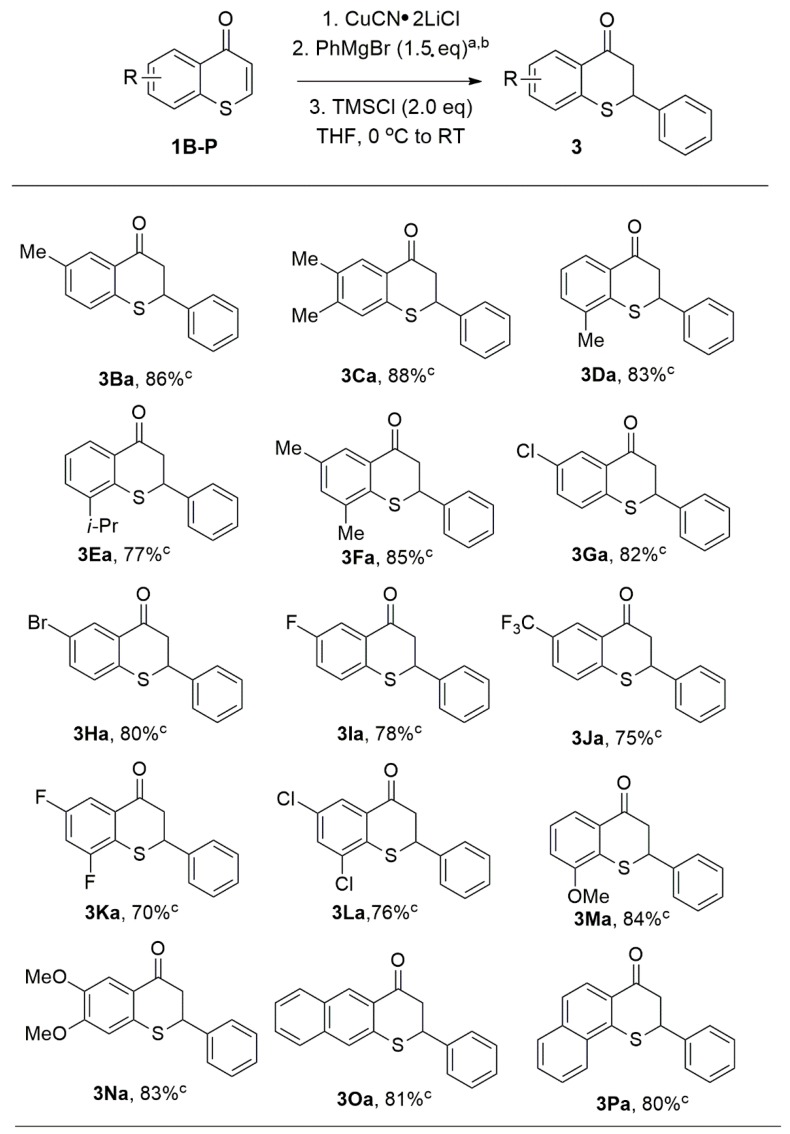
Reactions of PhMgBr with substituted thiochromones. a. All the reactions were performed using 1.5 equiv of PhMgBr in the presence of 2.0 equiv of TMSCI unless noted otherwise. b. PhMgBr were commercially available. c. Yields are based on isolated products by column chromatography.

The scope of thiochromones was also explored with PhMgBr to synthesize various thioflavanones. Phenylmagnesium bromide underwent conjugate addition to thiochromones **1B**–**1P** smoothly to furnish thioflavanones with good yields (Scheme 4). It was found that thiochromones with simple alkyl groups, such as methyl, on the aromatic ring reacted smoothly with PhMgBr to afford 1,4-adduct in good yields (Scheme 4, 83–88%). Bulky groups (*i*-Pr) were also tolerated (Scheme 4, **3Ea**, 77%). PhMgBr also underwent conjugate addition to thiochromones with halides F, Br, and Cl on the aromatic ring with high efficiency (Scheme 4, 78–82%). Thiochromones with two halides on the aromatic ring, such as 6,8-diflurothiochromanone and 6,8-dichlorothiochromone, were also tolerated to afford good yields of 1,4-adduct (Scheme 4, **3Ka**, 70% and **3La**, 76%). Electron-donating groups, such as MeO-, were also tolerated to furnish 1,4-adducts in an excellent yield (Scheme 4, **3Ma**, 84% and **3Na**, 83%). The steric hindrance was not a problem as PhMgBr adds to 8-substituted thiochromone (8-MeO-, 8-*i*-Pr) with high yields (Scheme 4). PhMgBr also underwent conjugate addition to thiochromones with extended aromatic structures with high efficiency (Scheme 4, **3Oa**, 81%; **3Pa**, 80%).

**Scheme 5:**
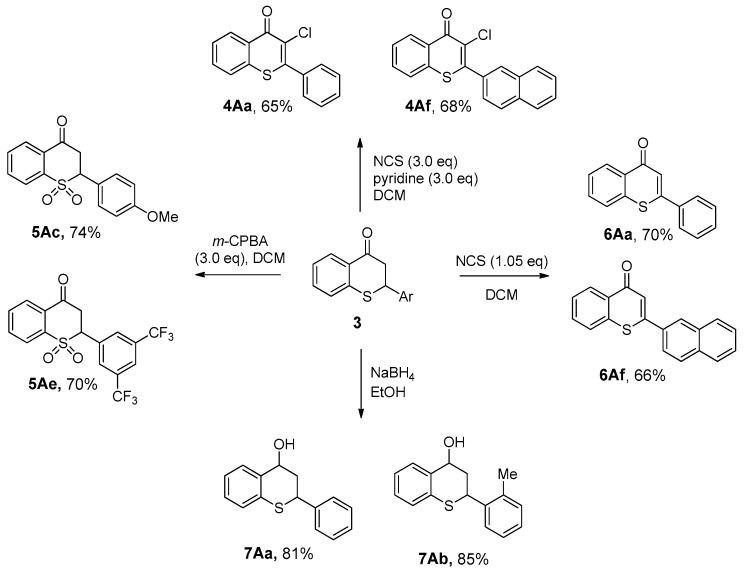
Synthetic applications of thioflavanones.

Thiochromanones, i.e., thiochroman-4-ones, 2-alkyltiochroman-4-ones, and thioflavanones (2-arylthioflavanones), are valuable synthons and vital precursors in organic synthesis for bioactive thiochromanone derivatives. [31,32,33,34,35,36,37,38] In our previous investigation, 2-alkylthiochroman-4-ones were successfully converted into other useful thiochroman-4-one derivatives for additional synthetic application [66]. The 1,4-adducts-thioflavones can also be utilized for further synthetic applications towards 2-aryl substituted thiochroman-4-one derivatives (Scheme 5). For example, thioflavanone **3** was converted to chlorinated thiochromone **4** upon treatment with excess *N*-chlorosuccinimide and pyridine (Scheme 5, **4Aa**, 65%; **4Af**, 68%). It was also oxidized to sulfone **5** with excess *m*-chloroperobenzoic acid (*m*-CPBA) in dichloromethane (Scheme 5, **5Ac**, 74%; **5Ae**, 70%). After treatment with *N*-chlorosuccinimide (NCS, 1.0 equivalent), thioflavanone **3** was transformed to thiochromone **6** with good yields (Scheme 5, **6Aa**, 70%; **6Af**, 66%). It was also reduced by sodium borohydride to the corresponding alcohol **7** (Scheme 3, **7Aa**, 81%; **7Ab**, 85%).

## 3. Materials and Methods

### 3.1. General Methods

The ^1^H, ^13^C, and ^19^F-NMR spectra were recorded on a BRUKER Ascend^TM^ 400 NMR spectrometer (Bruker Corporation, Billerica, MA, USA), operating at 400 MHz for ^1^H and 100 MHz for ^13^C and 376 MHz for ^19^F. Samples for NMR spectra were dissolved in deuterated chloroform unless otherwise noted. Analytical thin layer chromatography (TLC) was performed on silica gel plates, 60 mesh, with an F_254_ indicator. Visualization was accomplished by UV light (254 nm), and/or a 10% ethanol solution of phosphomolybdic acid and/or KMnO_4_ stain prepared by dissolving 1.5 g KMnO_4_, 10 g potassium carbonate, and 1.25 mL of 10% sodium hydroxide in 200 mL of water. Flash chromatography was performed with 230–400 mesh silica gel. Infrared (IR) spectra were recorded on a Nicolet iS10 FT-IR spectrometer (Thermo Scientific, Waltham, MA, USA) as neat samples (thin films).

### 3.2. Materials

Solvents and chemicals were obtained from commercial sources and used without further purification unless stated otherwise. Anhydrous tetrahydrofuran (THF) was purchased from Sigma Aldrich (Sigma-Aldrich, St. Louis, MO, USA). TMSCl was distilled from CaH_2_ under a positive N_2_ atmosphere. Grignard reagents were purchased from Sigma Aldrich or prepared from the corresponding bromocompounds. All glassware was flamed-dried under high vacuum and purged with argon to cool to room temperature. Low-temperature baths were prepared using dry ice-isopropanol slush bath mixtures. All 1,4-conjugate addition reactions with Grignard reagents were conducted under a positive dry argon atmosphere in anhydrous solvents in flasks fitted with a rubber septum.

### 3.3. General Procedure A: Conjugate Addition Reactions of Grignard Reagents (RMgX or ArMgX ; X = Cl or Br) to Thiochromones Catalyzed by CuCN∙2LiCl (0.2 eq)

To flame-dried LiCl (8.5 mg, 0.2 mmol, 0.4 equivalent) under argon, CuCN (9.0 mg, 0.1 mmol, 0.2 equivalent) and THF (1.0 mL) were added. The resultant mixture was stirred for 10 min at room temperature and then cooled to 0 °C followed by the addition of a Grignard reagent (0.75 mmol, 1.5 equivalent). The resultant solution was stirred for an additional 30 min at 0 °C under argon and then thiochromones [0.5 mmol mixed with TMSCl (1.0 mmol) in THF 1.0 mL)] were added. The reaction mixture was allowed to warm up to room temperature during overnight stirring. Then, the reaction mixture was quenched with saturated aqueous NH_4_Cl (10.0 mL) and extracted with ethyl acetate (3 × 10.0 mL). The combined organic phase was washed with brine (15.0 mL), dried over anhydrous Na_2_SO_4_, filtered, concentrated in vacuo, and purified by flash column chromatography (silica gel, 0–2% ethyl acetate in hexane, *v*/*v*) to give pure compounds.

### 3.4. Synthesis

HRMS data for compounds **2Ag**, **3Ag**, **2Ka**, **2La**, **2Na**, **3Ea**, **3Fa**, **3Ka**-**3Ma**, **3Pa**, **4Af**, **5Ac**, **5Ae**, and **7Ab** were analyzed by TOF MS. Compounds **2Aa**–**2Af** [66], **2Ah** [65], **2Ba**–**2Ia** [66], **2Ma** [66], **2Oa** [66], **3Aa**–**3Af** [51,67], **3Ah**–**3Ai** [51,59], **3Ba**–**3Da** [51,67], **3Ga**–**3Ja** [51,67], **3Ma** [51], **3Na[58]**, **3Oa** [51,67], **4Aa** [68], **6Aa** [69], **6Af** [70], and **7Aa** [59] have been fully characterized and reported. (Supplementary materials)

#### 3.4.1. Synthesis of 2-Cyclopropylthiochroman-4-one (**2Ag**)

Employing General Procedure A, using cyclopropylmagnesium bromide (1.0 M, 1.0 mL, 1.0 mmol) and thiochromone (81 mg, 0.5 mmol), after purification by flash column chromatography (silica gel, 0–2% ethyl acetate: hexanes, *v*/*v*), gave a light-yellow solid **2Ag** (84 mg, 82%): mp 83–84 °C; IR (neat) 3056 (w), 3001 (w), 2921 (w), 1671 (s), 1590 (s), 1456 (w), 1434 (s), 1394 (w), 1284 (s), 1229 (m), 1154 (w), 1068 (w), 1023 (w), 954 (w), 759 (s) cm^−1^; ^1^H NMR (400 MHz, CDCl_3_) δ 0.22–0.29 (m, 1 H), 0.29–0.35 (m, 1 H), 0.55–0.65 (m, 2 H), 0.95–1.07 (m, 1 H), 2.70 (ddd, *J* = 2.80, 9.60, 11.6 Hz, 1 H), 2.88 (dd, *J* = 11.6, 16.4 Hz, 1 H), 3.08 (dd, *J* = 2.8, 16.4 Hz, 1 H), 7.09 (ddd, *J* = 1.2, 7.2, 8.0 Hz, 1H), 7.20 (ddd, *J* = 0.4, 1.2, 8.0 Hz, 1H), 7.31 (ddd, *J* = 1.6, 7.2, 8.0 Hz, 1H), 8.01 (ddd, *J* = 0.4, 1.6, 8.0 Hz, 1H); ^13^C NMR (100 MHz, CDCl_3_) δ 4.7, 5.0, 15.4, 46.4, 47.2, 124.8, 127.5, 129.0, 130.6, 133.5, 142.1, 194.7; HRMS (EI-ion trap) *m*/*z*: [M + 1]^+^ calcd for C_12_H_13_OS, 205.0687; found 205.0679.

#### 3.4.2. Synthesis of 2-(9-Phenanthryl)thiochroman-4-one (**3Ag**)

Employing General Procedure A, using 9-phenanthrenylmagnesium bromide (1.0 M, 1.0 mL, 1.0 mmol) and thiochromone (81 mg, 0.50 mmol), after purification by flash column chromatography (silica gel, 0–2% ethyl acetate: hexanes, *v*/*v*), gave a yellow solid **3Ag** (133 mg, 78%): mp 180–181 °C; IR (neat) 3062 (w), 2922 (w), 1668 (s), 1582 (m), 1450 (w), 1439 (w), 1295 (m), 1089 (w), 892 (w), 760 (w), 746 (m), 722 (m) cm^−1^; ^1^H NMR (400 MHz, CDCl_3_) δ 3.38 (dd, *J* = 2.8, 16.4 Hz, 1 H), 3.56 (dd, *J* = 12.8, 16.4 Hz, 1 H), 5.44 (dd, *J* = 2.8, 12.8 Hz, 1 H), 7.19 (ddd, *J* = 1.2, 7.2, 8.0 Hz, 1H), 7.37 (ddd, *J* = 1.2, 7.2, 8.0 Hz, 1H), 7.54 (ddd, *J* = 0.8, 7.2, 8.0 Hz, 1H), 7.57–7.65 (m, 3H), 7.80-7.85 (m, 2H), 8.13–8.19 (m, 2H), 8.60 (d, *J* = 8.0 Hz, 1H), 8.69 (dd, *J* = 1.2, 8.0 Hz, 1 H); ^13^C NMR (100 MHz, CDCl_3_) δ 41.2, 46.2, 122.5, 123.6, 123.7, 125.4, 126.0, 126.9, 127.1, 127.4, 127.5, 129.0, 129.4, 129.5, 130.3, 130.5, 131.0, 131.04, 132.3, 133.7, 142.2, 194.9; HRMS (EI-ion trap) *m*/*z*: [M + 1]^+^ calcd for C_23_H_17_OS, 341.1000; found 341.1014.

#### 3.4.3. Synthesis of 6,8-Difluoro-2-*n*-butylthiochroman-4-one (**2Ka**)

Employing General Procedure A, using *n*-butylmagnesium chloride (1.71 M, 0.44 mL, 0.75 mmol) and thiochromone (99 mg, 0.50 mmol), after purification by flash column chromatography (silica gel, 0–2% ethyl acetate: hexanes, *v*/*v*), gave a light-yellow oil **2Ka** (93 mg, 73%): IR (neat) 3083 (w), 2958 (s), 2929 (s), 2858 (s), 1686 (s), 1611 (m), 1571 (m), 1437 (m), 1571 (m), 1437 (s), 1326 (s), 1281 (s), 1217 (w), 1112 (m), 997 (m) 878 (w), 856 (w), 641 (w), 620 (m) cm^−1^; ^1^H NMR (400 MHz, CDCl_3_) δ 0.85 (t, *J* = 7.2 Hz, 3 H), 1.22–1.33 (m, 2H), 1.34–1.47 (m, 2H), 1.67 (q, *J* = 7.8 Hz, 2 H), 2.75 (dd, *J* = 11.2, 16.4 Hz, 1 H), 2.99 (dd, *J* = 2.8, 16.4 Hz, 1 H), 3.36-3.46 (m, 1H), 6.93 (ddd, *J* = 2.8, 8.0, 9.2 Hz, 1H), 7.57 (ddd, *J* = 1.2, 2.8, 8.8 Hz, 1H); ^13^C NMR (100 MHz, CDCl_3_) δ 13.9, 22.3, 28.7, 34.1, 41.1, 45.9, 108.7 (t, *J* = 26 Hz), 110.9 (dd, *J* = 3.0, 22 Hz), 125.4 (dd, *J* = 3.0, 18 Hz), 132.6 (dd, *J* = 3.0, 7.0 Hz), 158.6 (dd, *J* = 11, 246 Hz), 159.7 (dd, *J* = 11, 246 Hz), 192.6; ^19^F NMR (376 MHz, CDCl_3_) δ −107.4 (dd, *J* = 6.4, 8.7 Hz), −114.1 (dt, *J* = 6.3, 8.3 Hz); HRMS (EI-ion trap) *m*/*z*: [M + Na]^+^ calcd for C_13_H_14_ONaSF_2_, 279.0631; found 279.0642.

#### 3.4.4. Synthesis of 6,8-Dichloro-2-*n*-butylthiochroman-4-one (**2La**)

Employing General Procedure A, using *n*-butylmagnesium chloride (1.71 M, 0.32 mL, 0.54 mmol) and thiochromone (83 mg, 0.36 mmol), after purification by flash column chromatography (silica gel, 0–2% ethyl acetate: hexanes, *v*/*v*), gave a light-yellow oil **2La** (80 mg, 77%): IR (neat) 3067 (w), 2956 (m), 2928 (m), 2858 (m), 1686 (s), 1571 (m), 1538 (w), 1465 (w), 1426 (w), 1397 (s), 1285 (w), 1244 (m), 1120 (w), 1053 (w), 873 (w), 812 (m) cm^−1^; ^1^H NMR (400 MHz, CDCl_3_) δ 0.85 (t, *J* = 7.2 Hz, 3 H), 1.23–1.33 (m, 2H), 1.35–1.46 (m, 2H), 1.68 (q, *J* = 7.2 Hz, 2 H), 2.72 (dd, *J* = 11.2, 16.4 Hz, 1 H), 2.96 (dd, *J* = 3.2, 16.4 Hz, 1 H), 3.34–3.43 (m, 1H), 7.43 (d, *J* = 2.4 Hz, 1H), 7.93 (d, *J* = 2.4 Hz, 1H); ^13^C NMR (100 MHz, CDCl_3_) δ 13.9, 22.3, 28.6, 34.1, 40.8, 44.8, 127.2, 130.6, 132.4, 132.7, 133.5, 139.7, 192.9; HRMS (EI-ion trap) *m*/*z*: [M − 1]^+^ calcd for C_13_H_13_OSCl_2_, 287.0064; found 287.0067.

#### 3.4.5. Synthesis of 6,7-Dimethoxyl-2-*n*-butylthiochroman-4-one (**2Na**)

Employing General Procedure A, using *n*-butylmagnesium chloride (1.71 M, 0.27 mL, 0.47 mmol) and thiochromone (69 mg, 0.31 mmol), after purification by flash column chromatography (silica gel, 0–2% ethyl acetate: hexanes, *v*/*v*), gave a light-yellow oil 2Na (74 mg, 85%): IR (neat) 3077 (w), 3000 (w), 2954 (s), 2927 (s), 2853 (s), 1660 (s), 1591 (s), 1498 (s), 1462 (m), 1436 (m), 1388 (s), 1352 (m), 1258 (s), 1210 (s), 1177 (m), 1146 (m), 1098 (w), 1033 (s), 872 (w), 796 (w) cm^−1^; ^1^H NMR (400 MHz, CDCl_3_) δ 0.84 (t, J = 7.2 Hz, 3 H), 1.22–1.31 (m, 2H), 1.32–1.43 (m, 2H), 1.64 (q, *J* = 7.2 Hz, 2 H), 2.66 (dd, *J* = 11.2, 16.4 Hz, 1 H), 2.92 (dd, *J* = 3.2, 16.4 Hz, 1 H), 3.35–3.46 (m, 1H), 3.82 (s, 3H), 3.84 (s, 3H), 6.61 (s, 1H), 7.51 (s, 1H); ^13^C NMR (100 MHz, CDCl_3_) δ 13.9, 22.3, 28.9, 34.1, 42.3, 46.0, 56.0, 56.2, 109.0, 110.2, 123.9, 135.6, 147.1, 153.8, 193.6; HRMS (EI-ion trap) *m*/*z*: [M + 1]^+^ calcd for C_15_H_21_O_3_S, 281.1211; found 281.1210.

#### 3.4.6. Synthesis of 6-Isopropyl-2-phenylthiochroman-4-one (**3Ea**)

Employing General Procedure A, using phenylmagnesium bromide (2.8 M, 0.25 mL, 0.71 mmol) and thiochromone (95 mg, 0.47 mmol), after purification by flash column chromatography (silica gel, 0–2% ethyl acetate: hexanes, *v*/*v*), gave a yellow solid **3Ea** (102 mg, 77%): mp 78–79 °C; IR (neat) 3056 (w), 2965 (s), 2927 (m), 1673 (s), 1576 (m), 1489 (w), 1451 (w), 1411 (m), 1286 (w), 1262 (s), 1147 (w), 1973 (w), 1042 (w), 780 (w), 766 (w), 731 (w), 698 (w) cm^−1^; ^1^H NMR (400 MHz, CDCl_3_) δ 1.24 (d, *J* = 6.8 Hz, 3 H), 1.31 (d, *J* = 6.8 Hz, 3 H), 3.23 (dd, *J* = 2.8, 16.4 Hz, 1 H), 3.26–3.34 (m, 1H), 3.35 (dd, *J* = 13.6, 16.4 Hz, 1 H), 4.66 (dd, *J* = 2.8, 13.6 Hz, 1 H), 7.25 (dd, *J* = 7.6, 15.2 Hz, 1H), 7.34–7.51 (m, 6H), 8.09 (dd, *J* = 1.6, 8.0 Hz, 1 H); ^13^C NMR (100 MHz, CDCl_3_) δ 22.7, 23.1, 30.3, 44.9, 46.3, 124.8, 127.2, 127.5, 128.5, 129.0, 130.3, 130.9, 138.7, 140.4,, 145.8, 195.1; HRMS (EI-ion trap) *m*/*z*: [M + 1]^+^ calcd for C_18_H_19_OS, 283.1157; found 283.1147.

#### 3.4.7. Synthesis of 6,8-Dimethyl-2-phenylthiochroman-4-one (**3Fa**)

Employing General Procedure A, using phenylmagnesium bromide (2.8 M, 0.13 mL, 0.35 mmol) and thiochromone (42.8 mg, 0.23 mmol), after purification by flash column chromatography (silica gel, 0–2% ethyl acetate: hexanes, *v*/*v*), gave a white solid **3Fa** (52.5 mg, 85%): mp 123–124 °C; IR (neat) 3041 (w), 2977 (w), 2922 (m), 2852 (w), 1670 (s), 1600 (m), 1456 (m), 1421 (w), 1371 (w), 1310 (m), 1280 (m), 1250 (m), 1125 (w), 871 (m), 781 (m), 706 (m) cm^−1^; ^1^H NMR (400 MHz, CDCl_3_) δ 2.09 (s, 3 H), 2.14 (s, 3 H), 2.98 (dd, *J* = 3.2, 16.4 Hz, 1 H), 3.11 (dd, *J* = 13.2, 16.4 Hz, 1 H), 4.45 (dd, *J* = 2.8, 13.2 Hz, 1 H), 6.98 (dd, *J* = 0.8, 1.6 Hz, 1 H), 7.12–7.23 (m, 3 H), 7.24–7.28 (m, 2 H), 7.68 (dd, *J* = 0.8, 1.6 Hz, 1 H); ^13^C NMR (100 MHz, CDCl_3_) δ 19.9, 20.7, 44.9, 46.3, 127.1, 127.5, 128.5, 129.0, 130.4, 134.1, 135.0, 136.1, 138.3, 138.8, 195.1; HRMS (EI-ion trap) *m*/*z*: [M + 1]^+^ calcd for C_17_H_17_OS, 269.1000; found 269.0999.

#### 3.4.8. Synthesis of 6,8-Difluoro-2-phenylthiochroman-4-one (**3Ka**)

Employing General Procedure A, using phenylmagnesium bromide (2.8 M, 0.16 mL, 0.44 mmol) and thiochromone (58 mg, 0.293 mmol), after purification by flash column chromatography (silica gel, 0–2% ethyl acetate: hexanes, *v*/*v*), gave a light-yellow solid **3Ka** (57 mg, 70%): mp 70–71 °C; IR (neat) 3076 (s), 3031 (w), 2959 (w), 2892 (w), 1685 (s), 1609 (m), 1571 (m), 1438 (m), 1322 (m), 1282 (m), 1130 (w), 995 (m), 879 (w), 772 (w), 697 (w) cm^−1^; ^1^H NMR (400 MHz, CDCl_3_) δ 3.01 (dd, *J* = 0.4, 3.2 Hz, 1 H), 3.14 (dd, *J* = 13.2, 16.4 Hz, 1 H), 4.48 (dd, *J* = 3.2, 13.2 Hz, 1 H), 6.83 (ddd, *J* = 2.8, 8.0, 8.8 Hz, 1H), 7.13–7.26 (m, 5H), 7.51 (ddd, *J* = 1.6, 2.8, 8.8 Hz, 1 H); ^13^C NMR (100 MHz, CDCl_3_) δ 45.0, 46.3, 108.9 (dd, *J* = 2.5, 2.6 Hz), 111.2 (dd, *J* = 3, 23 Hz), 125.8 (dd, *J* = 4, 19 Hz), 127.5, 128.9, 129.1, 132.4 (dd, *J* = 3.0, 7.0 Hz), 137.7, 158.3 (dd, *J* = 11, 247 Hz), 159.91 (dd, *J* = 11, 247 Hz), 192.3 (t, *J* = 3.0 Hz); ^19^F NMR (376 MHz, CDCl_3_) δ −107.2 (dd, *J* = 6.8, 8.3 Hz), −113.5 (dt, *J* = 7.5, 8.3 Hz); HRMS (EI-ion trap) *m*/*z*: [M − 1]^+^ calcd for C_15_H_9_OSF_2_, 275.0342; found 275.0345.

#### 3.4.9. Synthesis of 6,8-Dichloro-2-phenylthiochroman-4-one (**3La**)

Employing General Procedure A, using phenylmagnesium bromide (2.8 M, 0.08 mL, 0.225 mmol) and thiochromone (33.6 mg, 0.15 mmol), after purification by flash column chromatography (silica gel, 0–2% ethyl acetate: hexanes, *v*/*v*), gave a light-yellow solid **3La** (35 mg, 76%): mp 112–113 °C; IR (neat) 3067 (w), 2921 (s), 1682 (s), 1571 (m), 1539 (w), 1453 (w), 1397 (s), 1283 (w), 1241 (m), 1177 (w), 1121 (w), 1051 (w), 808 (w), 767 (w), 697 (m) cm^−1^; ^1^H NMR (400 MHz, CDCl_3_) δ 3.00 (dd, *J* = 3.2, 16.4 Hz, 1 H), 3.10 (dd, *J* = 13.2, 16.4 Hz, 1 H), 4.45 (dd, *J* = 3.2, 13.2 Hz, 1 H), 7.15–7.45 (m, 5 H), 7.32 (d, *J* = 2.4 Hz, 1 H), 7.86 (d, *J* = 2.4 Hz, 1 H); ^13^C NMR (100 MHz, CDCl_3_) δ 44.8, 45.3, 127.51, 127.53, 128.9, 129.2, 131.0, 132.1, 132.5, 133.7, 137.5, 140.0, 192.6; HRMS (EI-ion trap) *m*/*z*: [M − 1]^+^ calcd for C_15_H_9_OSCl_2_, 306.9751; found 306.9765.

#### 3.4.10. Synthesis of 8-Methoxy-2-phenylthiochroman-4-one (**3Ma**)

Employing General Procedure A, using phenylmagnesium bromide (2.8 M, 0.16 mL, 0.44 mmol) and thiochromone (55 mg, 0.29 mmol), after purification by flash column chromatography (silica gel, 0–2% ethyl acetate: hexanes, *v*/*v*), gave a light-yellow solid **3Ma** (66 mg, 84%): mp 147–148 °C; IR (neat) 3017 (w), 2972 (W), 2936 (w), 1673 (s), 1579 (m), 1559 (m), 1451 (m), 1420 (m), 1316 (m), 1254 (s), 1155 (w), 1055 (w), 1031 (s), 789 (m), 770 (m), 712 (w), 697 (m) cm^−1^; ^1^H NMR (400 MHz, CDCl_3_) δ 3.08 (dd, *J* = 2.8, 16.0 Hz, 1 H), 3.21 (dd, *J* = 13.6, 16 Hz, 1 H), 3.82 (s, 3 H), 4.54 (dd, *J* = 2.8, 13.6 Hz, 1 H), 6.91 (dd, *J* = 1.2, 8.0 Hz, 1 H), 7.09 (t, *J* = 8.0 Hz, 1 H), 7.22–7.32 (m, 3H), 7.33–7.38 (m, 2H), 7.71 (dd, *J* = 1.2, 8.0 Hz, 1 H); ^13^C NMR (100 MHz, CDCl_3_) δ 44.4, 46.0, 56.3, 114.2, 121.1, 124.8, 127.5, 128.5, 129.0, 131.3, 131.9, 138.7, 155.1, 194.6; HRMS (EI-ion trap) *m*/*z*: [M + 1]^+^ calcd for C_16_H_15_O_2_S, 271.0793; found 271.0794.

#### 3.4.11. Synthesis of 2,3-Dihydro-2-phenyl-4*H*-naphtho[1,2-*b*]thiopyran-4-one (**3Pa**)

Employing General Procedure A, using phenylmagnesium bromide (2.8 M, 0.19 mL, 0.53 mmol) and thiochromone (75 mg, 0.354 mmol), after purification by flash column chromatography (silica gel, 0–2% ethyl acetate: hexanes, *v*/*v*), gave a light-yellow solid **3Pa** (82 mg, 80%): mp 144.0–145.0 °C; IR (neat) 3057 (w), 3031 (w), 2020 (w), 1668 (w), 1594 (m), 1552 (w), 1450 (w), 1326 (m), 1310 (m), 1267 (m), 1245 (m), 1165 (w), 1076 (w), 810 (m), 771 (m), 746 (m) cm^−1^; ^1^H NMR (400 MHz, CDCl_3_) δ 3.21 (dd, *J* = 2.8, 16.4 Hz, 1 H), 4.35 (dd, *J* = 13.6, 16.4 Hz, 1 H), 4.74 (dd, *J* = 3.2, 13.6 Hz, 1 H), 7.28–7.39 (m, 3H), 7.41–7.46 (m, 1H), 7.46–7.51 (m, 1H), 7.52–7.58 (m, 1H), 7.73–7.78 (m, 1H), 8.10–8.17 (m, 2H); ^13^C NMR (100 MHz, CDCl_3_) δ 45.4, 45.8, 124.1, 125.1, 125.4, 126.8, 127.6, 127.7, 128.6, 128.7, 129.1, 129.2, 130.1, 135.5, 138.4, 142.9, 194.6; HRMS (EI-ion trap) *m*/*z*: [M + 1]^+^ calcd for C_19_H_15_OS, 291.0844; found 291.0847.

#### 3.4.12. Synthesis of 3-Chloro-2-(2-naphthyl)-4*H*-thiochromen-4-one (**4Af**)

To a dichloromethane (DCM) solution of 2-(2-naphthyl)thiochroman-4-one (1.0 equivalent, 0.542 mmol, 157.2 mg), NCS (*N*-chlorosuccinimide) (3.0 equivalent, 1.63 mmol, 217 mg) was added. The reaction mixture was stirred at room temperature overnight (12 h). It was then quenched with water (10 mL) and extracted with DCM (3 × 10 mL). The organic layers were combined and dried over anhydrous Na_2_SO_4_. It was filtered and concentrated in vacuum. The crude product was purified by flash column chromatography (silica gel, 5 % ethyl acetate: hexanes, *v*/*v*) to give **4Af** as a white solid (119 mg, 68%): mp 161.0–162.0 °C; IR (neat) 3052 (w), 2922 (w), 1621 (s), 1586 (s) 1562 (m), 1500 (w), 1462 (w), 1435 (w), 1320 (w), 1305 (w), 1272 (w), 1135 (w), 1112 (w), 851 (m), 839 (m), 735 (m) cm^−1^; ^1^H NMR (400 MHz, CDCl_3_) δ 7.48–7.63 (m, 6 H), 7.82–7.88 (m, 2 H), 7.90 (d, *J* = 8.4 Hz, 1 H), 7.96 (t, *J* = 0.8 Hz, 1 H), 8.57 (ddd, *J* = 0.8, 1.6, 8.0 Hz, 1 H); ^13^C NMR (100 MHz, CDCl_3_) δ 125.6, 125.9, 126.5, 127.0, 127.7, 127.9, 128.1, 128.5, 128.6, 128.7, 129.8, 130.5, 131.9, 132.6, 132.8, 133.7, 136.6, 148.4, 174.8; HRMS (EI-ion trap) *m*/*z*: [M + 1]^+^ calcd for C_19_H_12_OSCl, 323.0297; found 323.0296.

#### 3.4.13. Synthesis of 2-(4-Methoxyphenyl)thiochroman-4-one 1,1-dioxide (**5Ac**)

To a dry DCM solution of 2-(4-methoxyphenyl)thiochroman-4-one (94 mg, 0.348 mmol) under Ar atmosphere in a 50-mL RB flask, excess 3-meta-chloroperoxybenzoic acid (*m*-CPBA, 3.0 equivalent, 180 mg, 1.04 mmol) was added. The resultant mixture was stirred at room temperature until the reaction was complete by TLC monitoring (4–5 h). Then the reaction mixture was quenched with NaHCO_3_ (10 mL) and diluted with DCM (10 mL). The organic layers were separated and the aqueous layer was extracted with DCM (2 × 10 mL). The organic layers were combined and washed with brine and dried over anhydrous Na_2_SO_4_. It was filtered and concentrated in vacuum. The crude product was purified by flash column chromatography (silica gel, 20% ethyl acetate: hexanes, *v*/*v*) to give a white solid **5Ac** (78 mg, 74%): mp 163–164 °C; IR (neat) 3100 (w), 2902 (w), 1690 (s), 1587 (w), 1376 (m), 1312 (m), 1273 (s), 1150 (m), 1115 (s), 934 (m), 904 (m), 759 (m) cm^−1^; ^1^H NMR (400 MHz, CDCl_3_) δ 3.41 (dd, *J* = 3.2, 17.6 1 H), 3.85 (s, 3 H), 3.95 (dd, *J* = 12.8, 17.6 Hz, 1 H), 4.85 (dd, *J* = 3.2, 12.8 Hz, 1 H), 7.00 (dd, *J* = 2, 6.8 Hz 1 H), 7.42 (dd, *J* = 2, 6.8 Hz, 2 H), 7.78 (td, *J* = 1.2, 8 Hz, 1 H), 7.85 (td, *J* = 1.2, 8.0 Hz, 1 H), 8.10 (dd, *J* = 1.2, 8.0 Hz, 1 H), 8.19 (dd, *J* = 1.2, 8 Hz, 1 H); ^13^C NMR (100 MHz, CDCl_3_) δ 43.2, 55.4, 63.5, 114.6, 119.7, 124.5, 128.7, 130.6, 131.1, 133.3, 135.1, 141.5, 160.9, 191.1; HRMS (EI-ion trap) *m*/*z*: [M + 1]^+^ calcd for C_16_H_15_O_4_S, 303.0691; found 303.0699.

#### 3.4.14. Synthesis of 2-[3,5-(Trifluoromethyl)phenyl]thiochroman-4-one 1,1-dioxide (**5Ae**)

To a dry DCM solution of 2-[3,5-(trifluoromethyl)phenyl]thiochroman-4-one (116 mg, 0.308 mmol) under Ar atmosphere in a 50-mL RB flask, excess 3-meta-chloroperoxybenzoic acid (mCPBA, 3.0 equivalent, 0.924 mmol, 159 mg, mCPBA was 70–75%) was added. The resultant mixture was stirred at room temperature until the reaction was complete by TLC monitoring (4–5 h). Then, the reaction mixture was quenched with NaHCO_3_ (10 mL) and diluted with DCM (10 mL). The organic layers were separated and the aqueous layer was extracted with DCM (2 × 10 mL). The organic layers were combined and washed with brine and dried over anhydrous Na_2_SO_4_. It was filtered and concentrated in vacuum. The crude product was purified by flash column chromatography (silica gel, 20% ethyl acetate: hexanes, *v*/*v*) to give a white solid **5Ae** (88 mg, 70%): mp 162–163 °C; IR (neat) 3100 (w), 2902 (w), 1690 (s), 1627 (w), 1587 (w), 1469 (w), 1376 (m), 1313 (s), 1273 (s), 1199 (m), 1115 (s), 1038 (w), 934 (m), 904 (m), 844 (w), 780 (w), 759 (s), 706 (w), 679 (w) cm^−1^; ^1^H NMR (400 MHz, CDCl_3_) δ 3.36 (dd, *J* = 3.2, 17.6 1 H), 3.92 (dd, *J* = 13.2, 17.6 Hz, 1 H), 4.94 (dd, *J* = 3.2, 12.8 Hz, 1 H), 7.74 (dt, *J* = 1.6, 7.6 Hz, 1 H), 7.89 (s, 1 H), 7.93 (s, 1 H), 8.01 (dd, *J* = 1.2, 8.0 Hz, 1 H), 8.13 (dd, *J* = 1.2, 8.0 Hz, 1 H); ^13^C NMR (100 MHz, CDCl_3_) δ 42.6, 63.1, 121.5, 122.8 (q, *J* = 258 Hz), 124.2, 124.5, 129.1, 130.0, (d, *J* = 3 Hz), 130.3, 130.7, 132.6 (q, *J* = 34 Hz), 133.9, 135.4, 140.7, 189.4; ^19^F NMR (376 MHz, CDCl_3_) δ −62.9; HRMS (EI-ion trap) *m*/*z*: [M + 1]^+^ calcd for C_17_H_11_O_3_SF_6_, 409.0333; found 409.0333.

#### 3.4.15. Synthesis of 2-(2-Methylphenyl)thiochroman-4-ol (**7Ab**)

To a dry ethanol solution (3.0 mL) of 2-(2-methylphenyl)thiochroman-4-one (50 mg, 1.0 eq, 0.197 mmol, 50 mg) under argon, sodium borohydride (5 mg, 0.6 eq, 0.12 mmol, 10 mg) was added portion-wise. The resultant mixture was stirred at room temperature for 2 h when TLC monitoring showed that all the starting material was gone. Then solvent was evaporated, ice water (10 mL) was added, and the mixture was acidified with 10% HCl to pH = 1–2. It was then extracted with ethyl acetate (3 × 10 mL) and organic layers were combined, and washed with brine (15 mL). The organic layer was dried (Na_2_SO_4_), filtered, and evaporated under vacuum to give the crude product. The crude product was then purified by flash column chromatography (silica gel, 10% ethyl acetate: hexanes, *v*/*v*) to give 2-(2-methylphenyl)thiochroman-4-ol **7Ab** as a white solid (41 mg, 81%): mp 146–147 °C; IR (neat) 3269 (sb), 3064 (w), 3021 (w), 2918 (s), 1953 (w), 1916 (w), 1590 (w), 1565 (w), 1487 (m), 1486 (s), 1435 (m), 1244 (w0, 1220 (w), 1063 (m), 1021 (m), 753 (s) cm^−1^; ^1^H NMR (400 MHz, CDCl_3_) δ 2.0 (s, 1H), 2.47 (s, 3H), 2.48–2.53 (m, 1H), 2.67 (ddd, *J* = 2.8, 5.2, 12.8 Hz, 1 H), 4.86 (dd, *J* = 3.2, 12 Hz, 1 H), 5.0–5.10 (m, 1 H), 7.11–7.28 (m, 6H), 7.49–7.55 (m, 1 H), 7.65–7.73 (m, 6H); ^13^C NMR (100 MHz, CDCl_3_) δ 19.3, 40.0, 40.3, 70.0, 124.8, 125.9, 126.7, 126.79, 126.80, 127.7, 127.8, 130.7, 134.1, 135.9, 136.6, 138.4; HRMS (EI-ion trap) *m*/*z*: [M + Na]^+^ calcd for C_16_H_16_ONaS, 279.0820; found 279.0826.

## 4. Conclusions

In conclusion, we successfully developed the conjugate addition of Grignard reagents to thiochromones catalyzed by CuCN∙2LiCl in the presence of chlorotrimethylsilane (TMSCl) as an activator to afford 2-alkylthiochroman-4-ones and thioflavanones (2-arylthiochromon-4-ones) in good yields. This reaction was shown to work well with a broad range of Grignard reagents, both alkyl and aromatic Grignard reagents. This synthetic approach will allow access to a broader scope of thiochromanones and thioflavanones due to the readily available Grignard reagents compared to organolithium reagents. Very interesting compounds, such as 6,8-difluorosubstituted thiochroman-4-ones, were synthesized for further studies. The 1,4-adducts, both 2-alkylthiochroman-4-ones and thioflavanones, can be utilized for additional synthetic applications and provide quick access that privileges sulfur-heterocycles.

## Supplementary Materials

The following are available online.
