# Supplementary material for: Conjugate Addition of Grignard Reagents to Thiochromones Catalyzed by Copper Salts: A Unified Approach to Both 2-Alkylthiochroman-4-One and Thioflavanone
Source: Molecules. 2020 May 1;25(9):2128. doi: 10.3390/molecules25092128 (PMC7248974; doi:10.3390/molecules25092128)

Conjugate addition of Grignard reagents to thiochromones catalyzed by copper salts: a unified approach to both 2-alkylthiochroman-4-one and thioflavanone

Tania J. Bellinger [1], Teavian Harvin[1], Ti'Bran Pickens-Flynn[1], Nataleigh Austin[1], Samuel H. Whitaker[1], Mai Ling C. Tang Yuk Tutein[1], Dabria T. Hulkins[1], Nichele Deese[1], and Fenghai Guo [1, 2,*]

[1] Department of Chemistry, Winston Salem State University, 601 S. Martin Luther King Jr. Dr., Winston Salem, NC 27110, USA;
[2] Biomedical Research Infrastructure Center, Winston Salem State University, Winston Salem, NC 27110, USA;
* Correspondence: guof@wssu.edu; Tel.: +01-336-750-3158

Received: date; Accepted: date; Published: date

Supporting Information

Table of contents

^{1}H, ^{13}C-NMR, and ^{19}F spectra:

^{1}H, ^{13}C-NMR spectra for compounds: **2Ag, 3Ag, 2Ka, 2La, 2Na**, **3Ea**, **3Fa**, **3Ka**-**3Ma**, **3Pa**, **4Af**, **5Ac**, **5Ae**, **and 7Ab**……………………………………………………………………………………………S2-4, S7-11, S14-19, and S21

^{19}F spectra for compounds: **2Ka**, **3Ka** and **5Ae** ………………………………………………………… S5-6, S12-13, and S20

2Ag

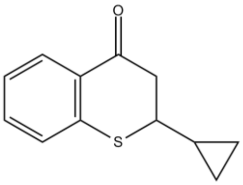
O
S

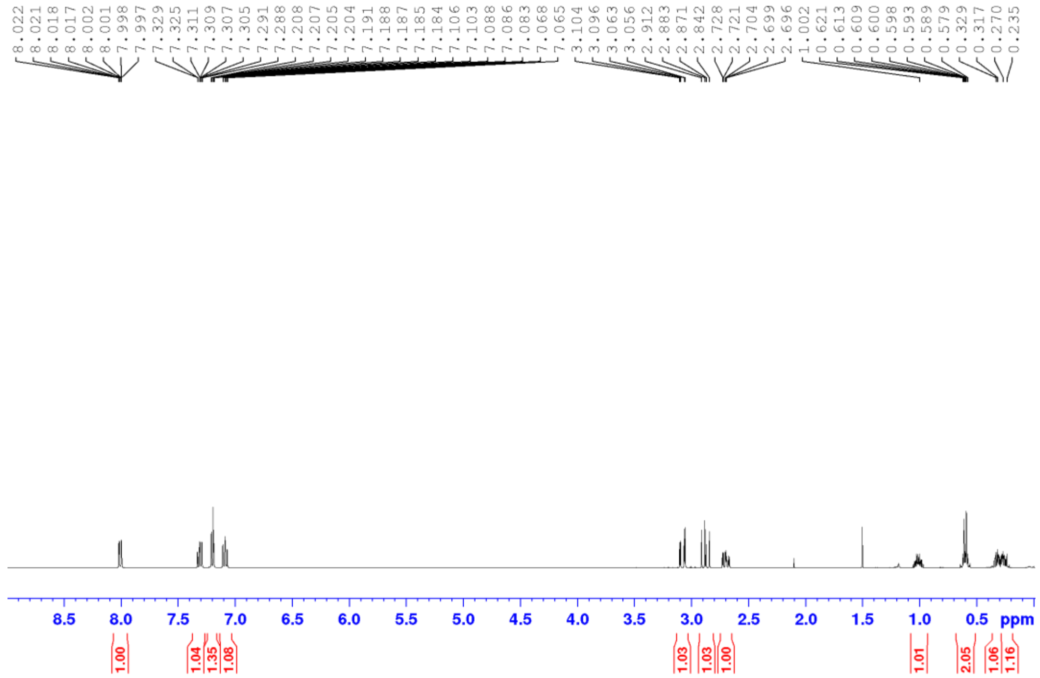
8.022 8.021 8.018 8.017 8.002 8.001 7.998 7.997 7.329 7.325 7.311 7.309 7.307 7.305 7.291 7.288 7.208 7.207 7.205 7.204 7.191 7.188 7.187 7.185 7.184 7.106 7.103 7.088 7.086 7.083 7.068 7.065 3.104 3.096 3.063 3.056 2.912 2.883 2.871 2.842 2.728 2.721 2.704 2.699 2.696 1.002 0.621 0.613 0.609 0.600 0.598 0.593 0.589 0.579 0.329 0.317 0.270 0.235
8.5 8.0 7.5 7.0 6.5 6.0 5.5 5.0 4.5 4.0 3.5 3.0 2.5 2.0 1.5 1.0 0.5 ppm
1.00 1.04 1.35 1.08 1.03 1.03 1.00 1.01 2.05 1.06 1.16

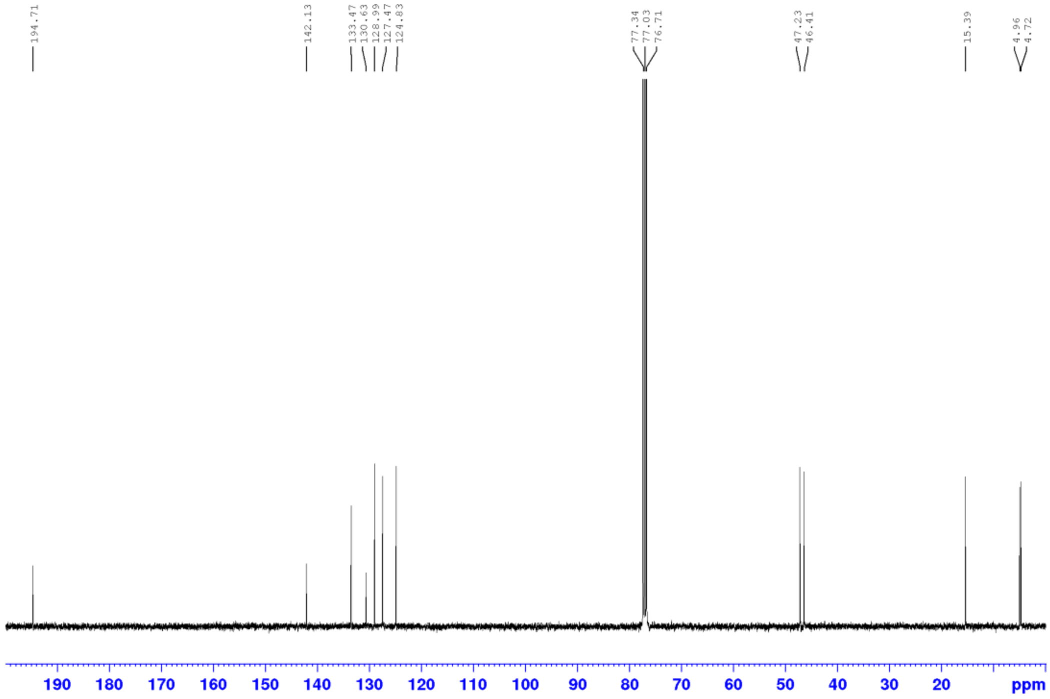
194.71 142.13 133.47 130.63 128.99 127.47 124.83 77.34 77.03 76.71 47.23 46.41 15.39 4.96 4.72
190 180 170 160 150 140 130 120 110 100 90 80 70 60 50 40 30 20 ppm

3Ag

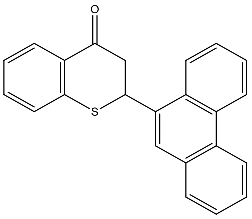
O
S

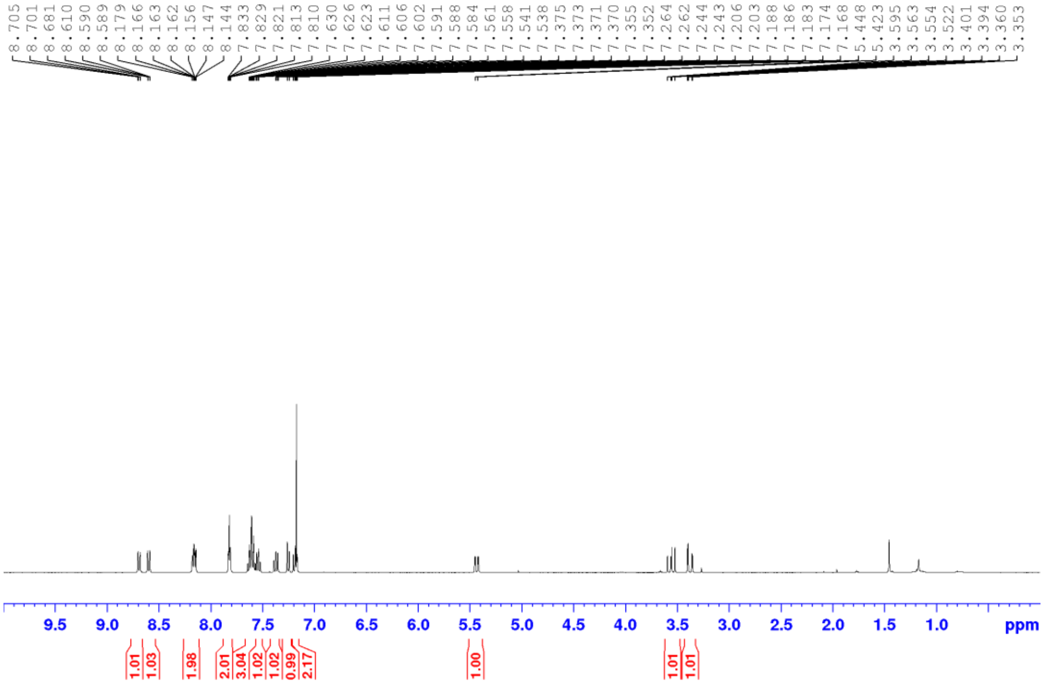
8.705
8.701
8.681
8.610
8.590
8.589
8.179
8.166
8.163
8.162
8.156
8.147
8.144
7.833
7.829
7.821
7.813
7.810
7.630
7.626
7.623
7.611
7.606
7.602
7.591
7.588
7.584
7.561
7.558
7.541
7.538
7.375
7.373
7.371
7.370
7.355
7.352
7.264
7.262
7.244
7.243
7.206
7.203
7.188
7.186
7.183
7.174
7.168
5.448
5.423
3.595
3.563
3.554
3.522
3.401
3.394
3.360
3.353
9.5
9.0
8.5
8.0
7.5
7.0
6.5
6.0
5.5
5.0
4.5
4.0
3.5
3.0
2.5
2.0
1.5
1.0
ppm
1.01
1.03
1.98
2.01
3.04
1.02
1.02
0.99
2.17
1.00
1.01
1.01

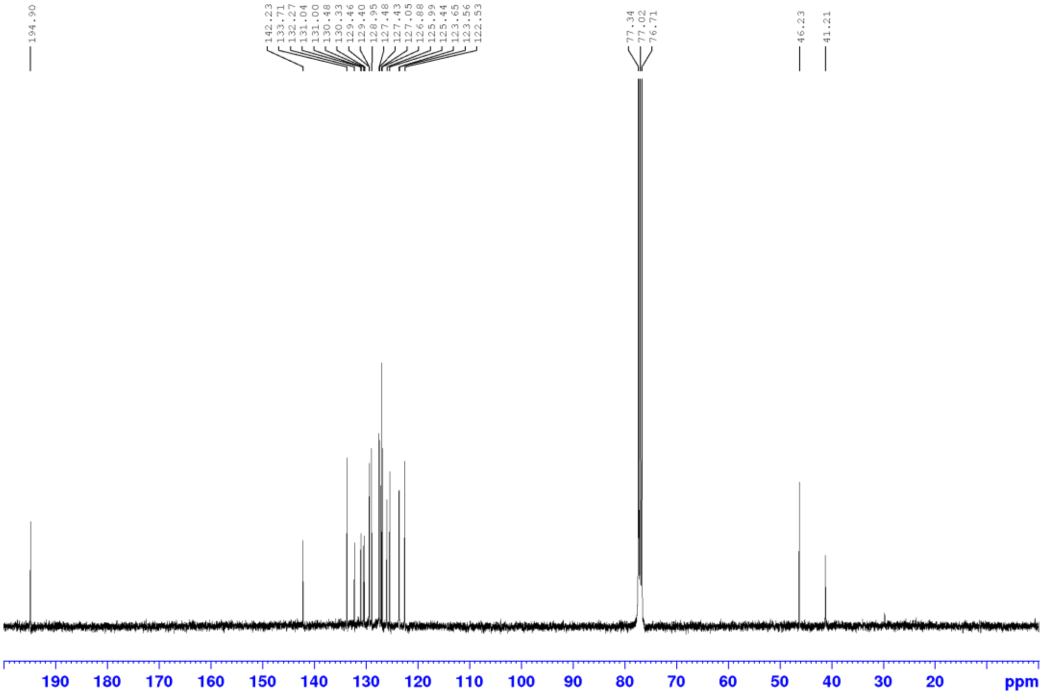
194.90
142.23
133.71
132.27
131.04
131.00
130.48
130.33
129.46
129.40
128.95
127.48
127.43
127.05
126.88
125.99
125.44
123.65
123.56
122.53
77.34
77.02
76.71
46.23
41.21
190
180
170
160
150
140
130
120
110
100
90
80
70
60
50
40
30
20
ppm

2Ka

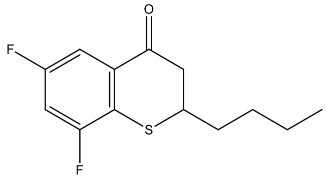

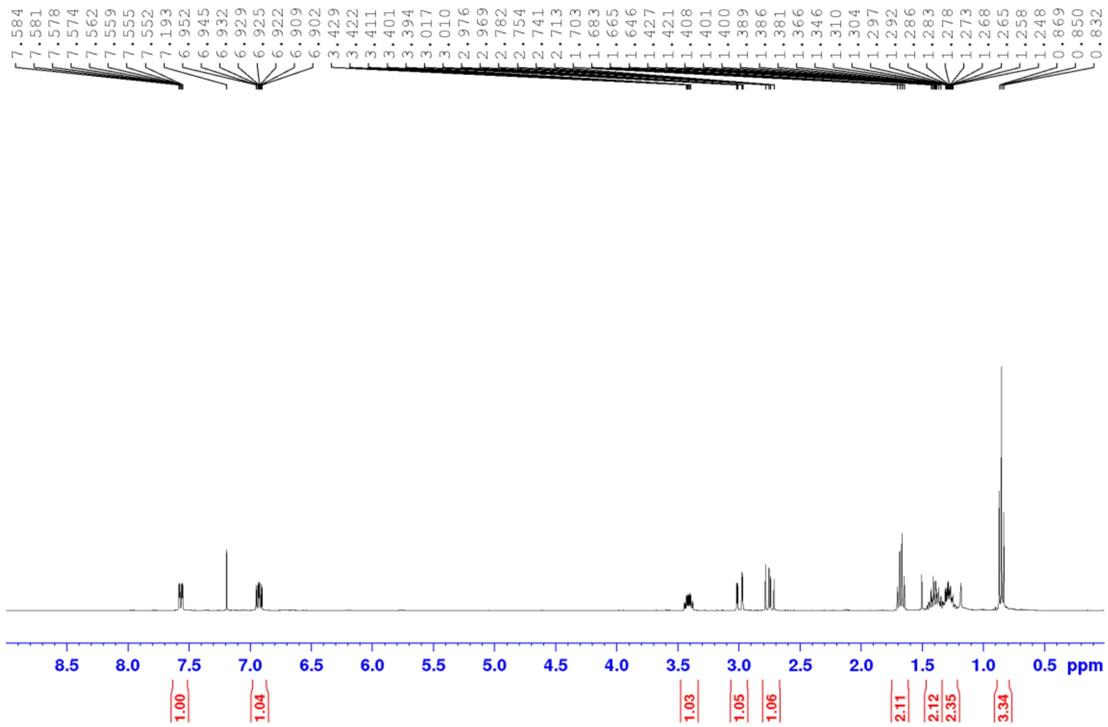

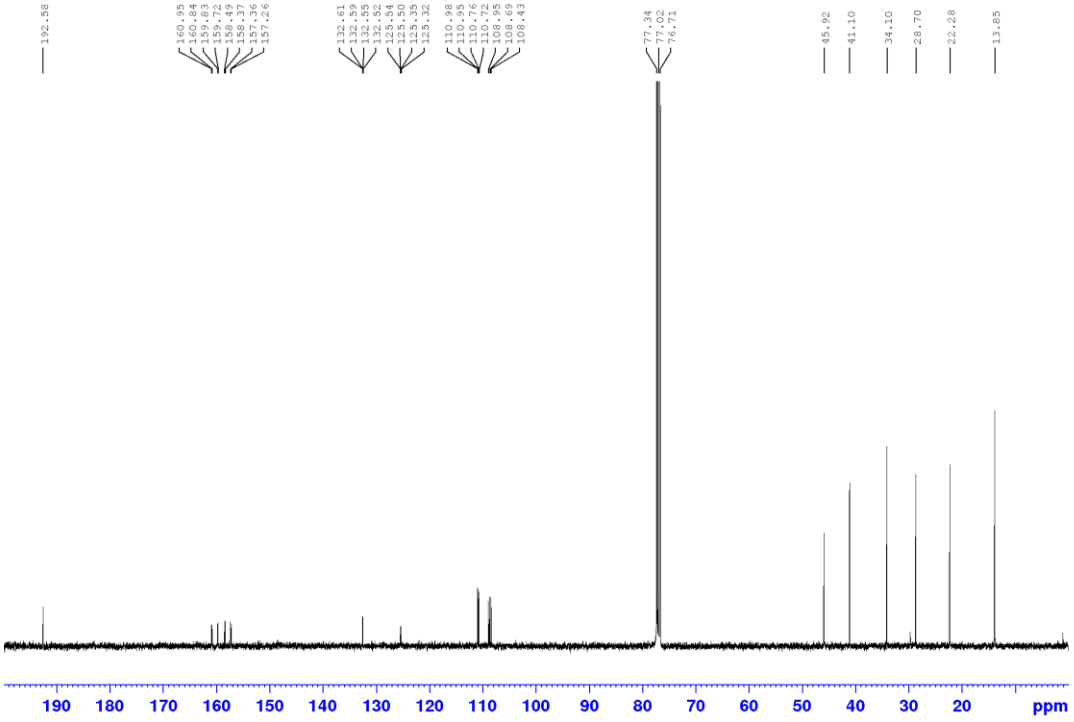

2Ka

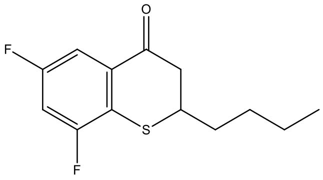
O
F
S
F

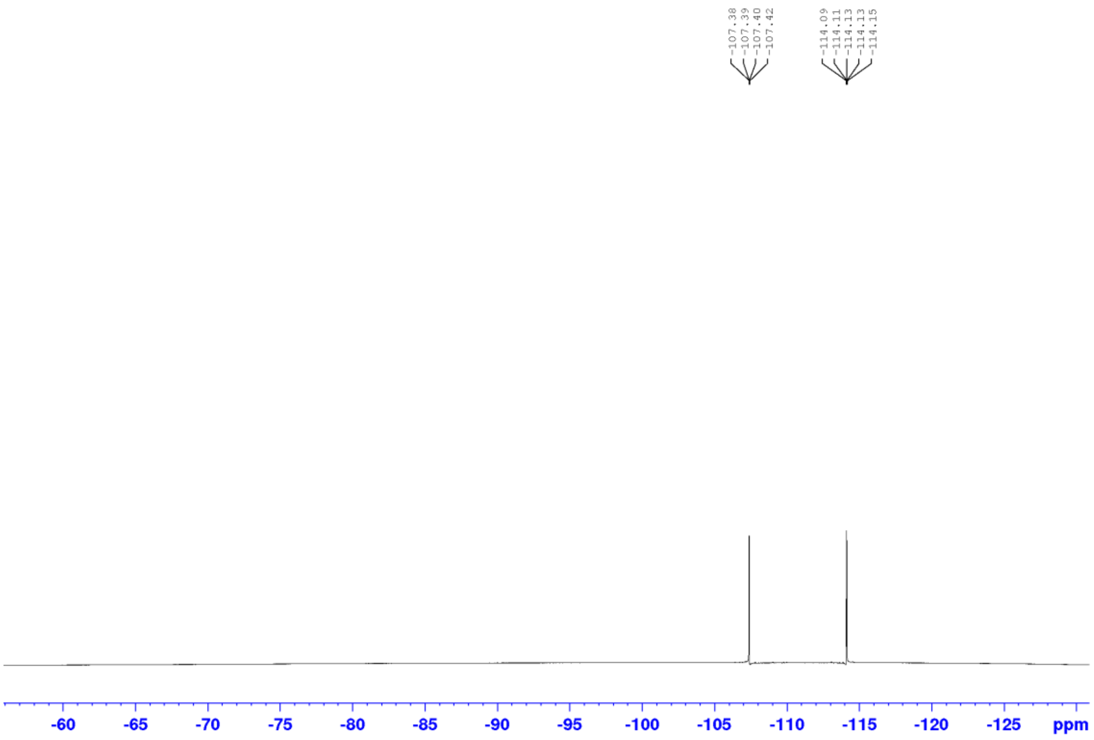
-107.38
-107.39
-107.40
-107.42
-114.09
-114.11
-114.13
-114.13
-114.15
-60
-65
-70
-75
-80
-85
-90
-95
-100
-105
-110
-115
-120
-125
ppm

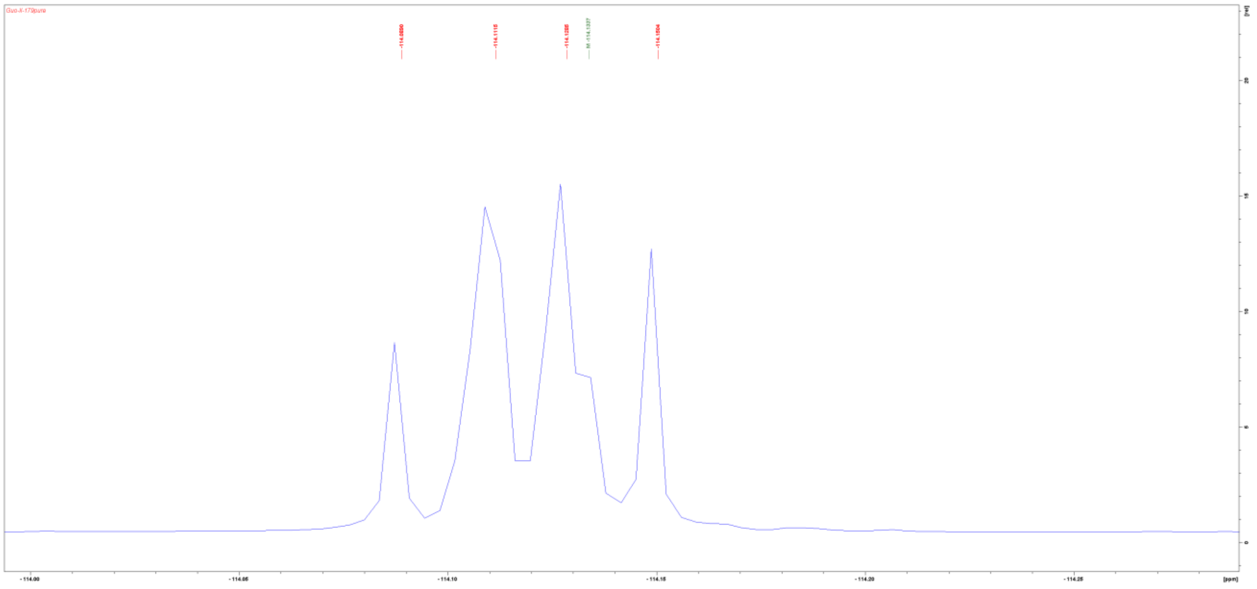

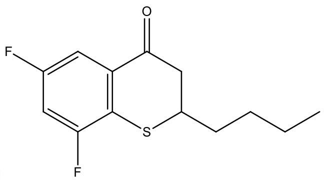
O
F
S
F

2Ka

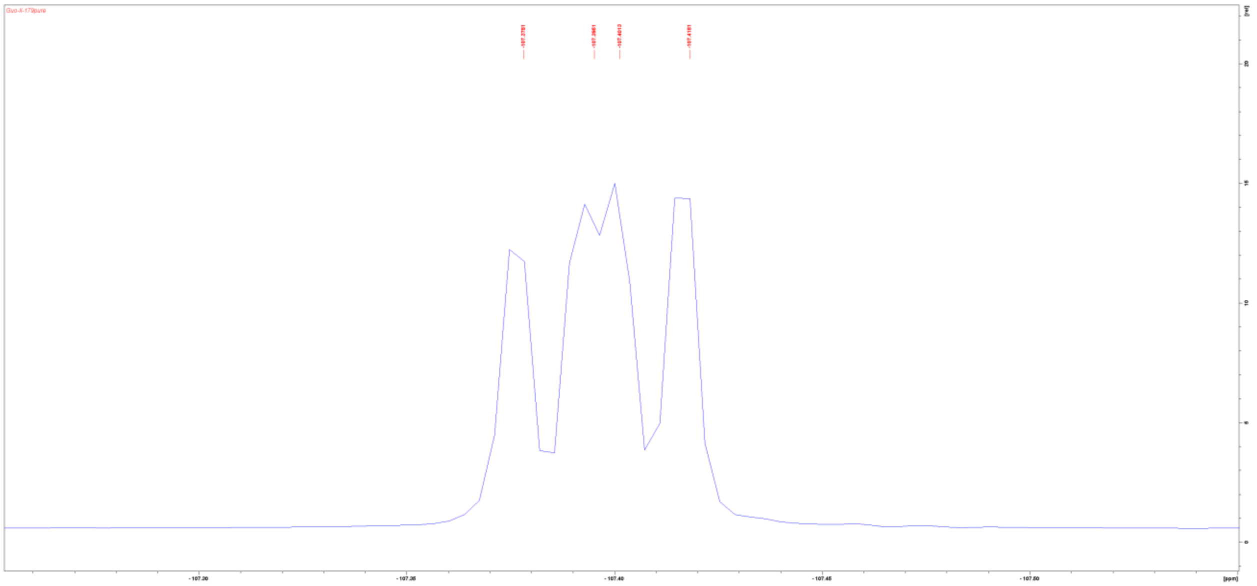

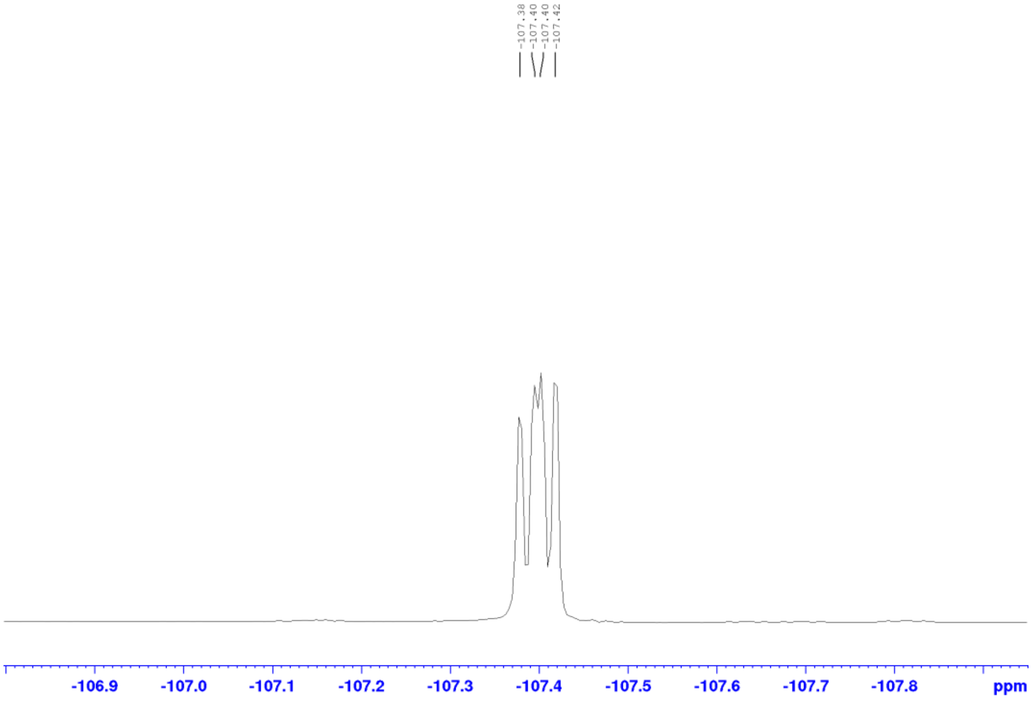
-107.38
-107.40
-107.40
-107.42
-106.9
-107.0
-107.1
-107.2
-107.3
-107.4
-107.5
-107.6
-107.7
-107.8
ppm

2La

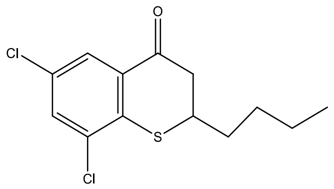
O
Cl
S
Cl

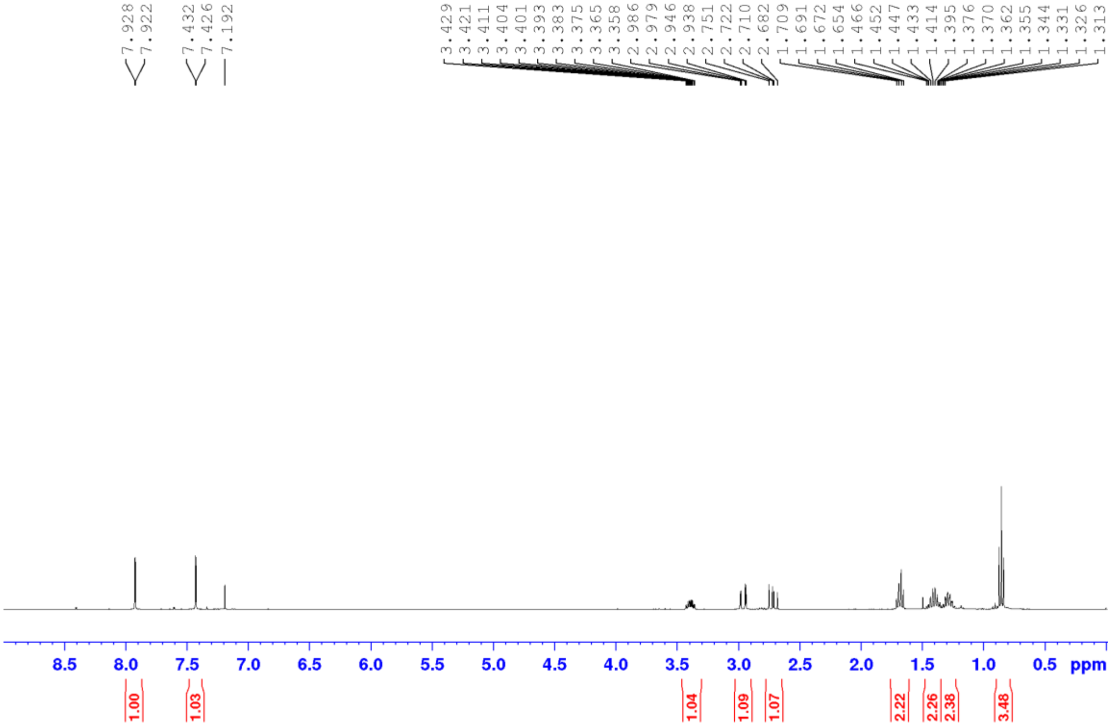
7.928
7.922
7.432
7.426
7.192
3.429
3.421
3.411
3.404
3.401
3.393
3.383
3.375
3.365
3.358
2.986
2.979
2.946
2.938
2.751
2.722
2.710
2.682
1.709
1.691
1.672
1.654
1.466
1.452
1.447
1.433
1.414
1.395
1.376
1.370
1.362
1.355
1.344
1.331
1.326
1.313
8.5 8.0 7.5 7.0 6.5 6.0 5.5 5.0 4.5 4.0 3.5 3.0 2.5 2.0 1.5 1.0 0.5 ppm
1.00
1.03
1.04
1.09
1.07
2.22
2.26
2.38
3.48

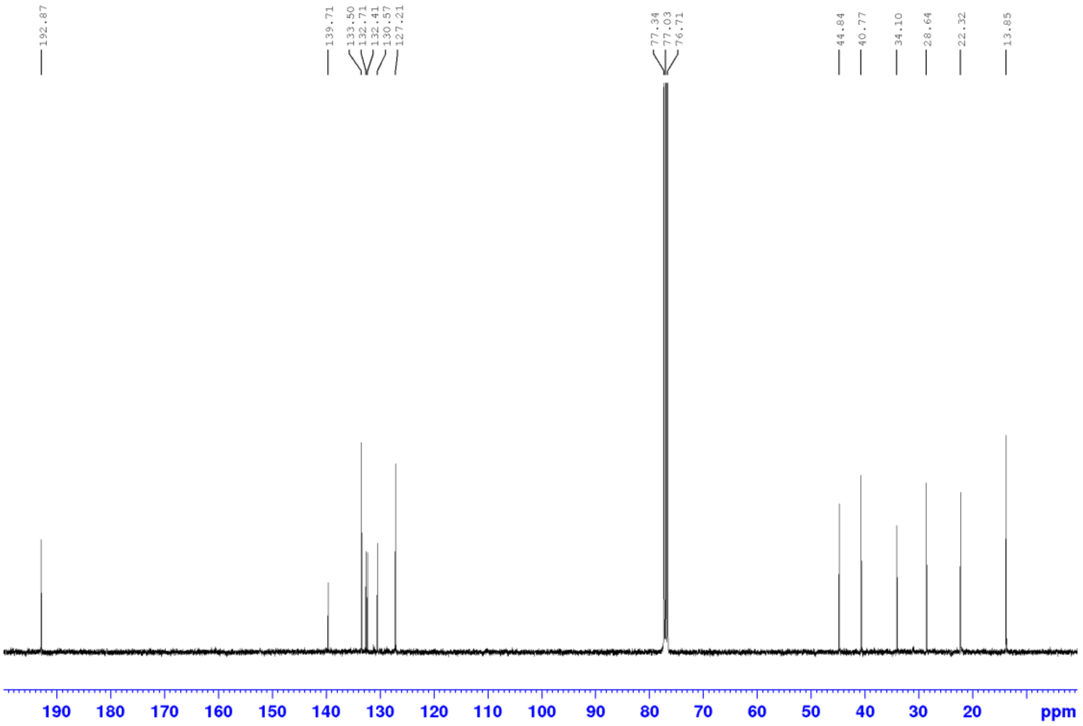
192.87
139.71
133.50
132.71
132.41
130.57
127.21
77.34
77.03
76.71
44.84
40.77
34.10
28.64
22.32
13.85
190 180 170 160 150 140 130 120 110 100 90 80 70 60 50 40 30 20 ppm

2Na

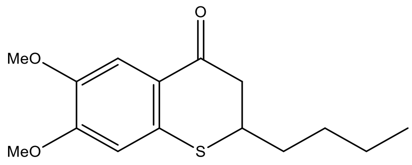

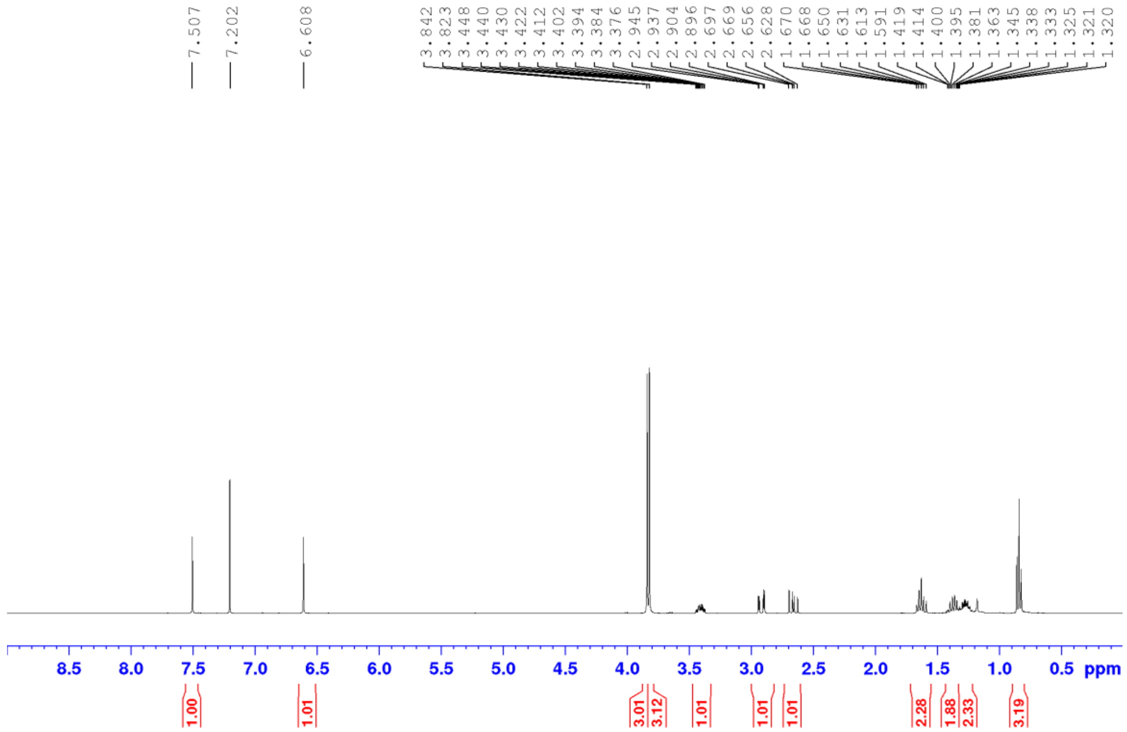

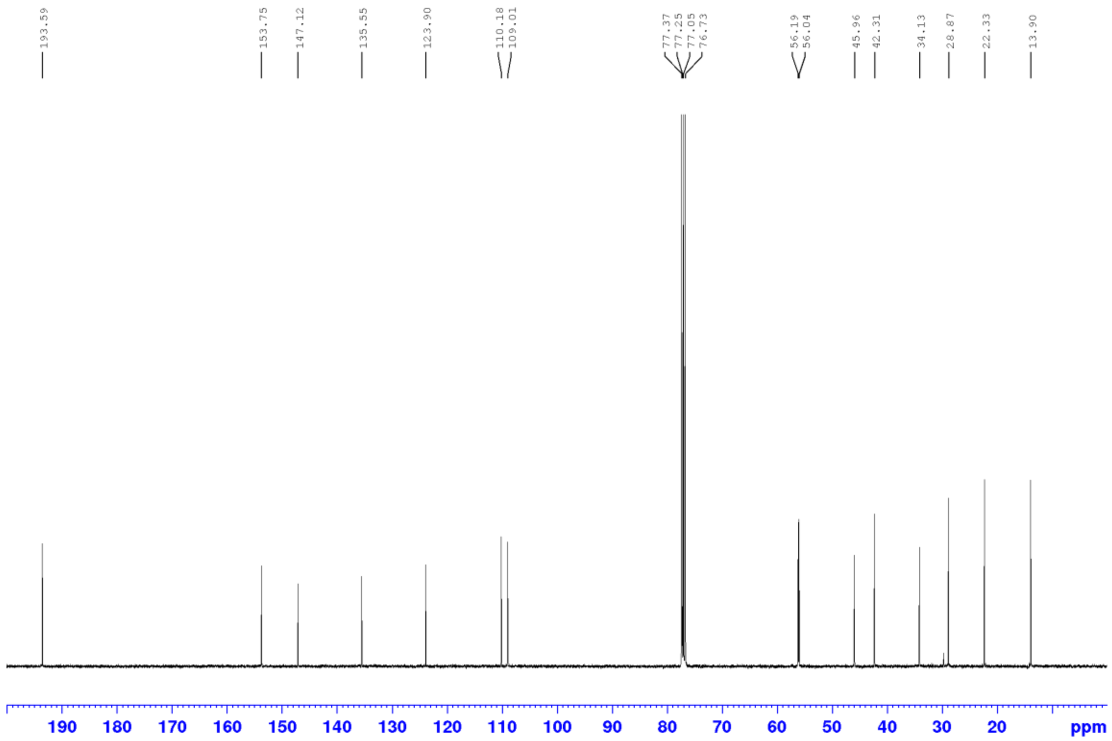

3Ea

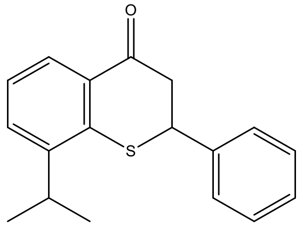

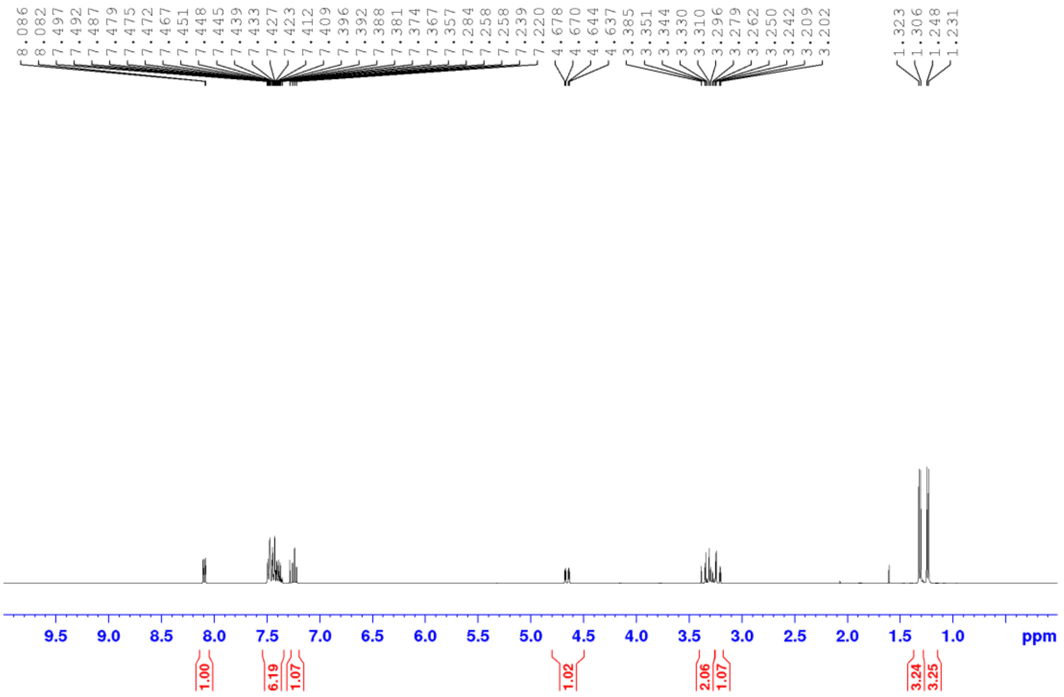

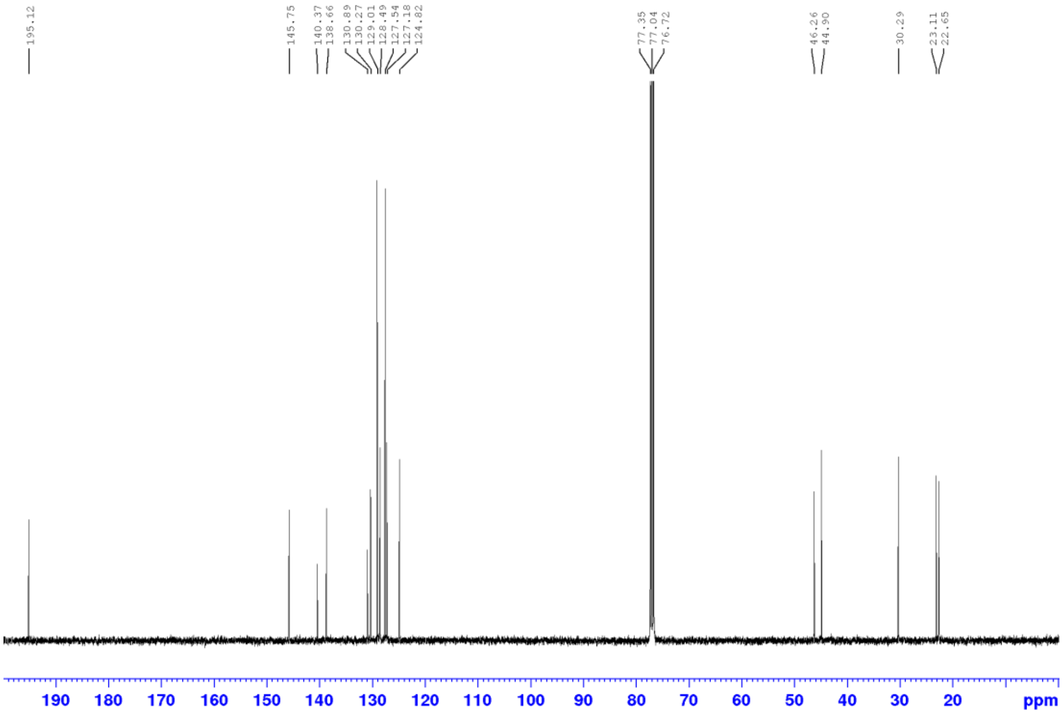

3Fa

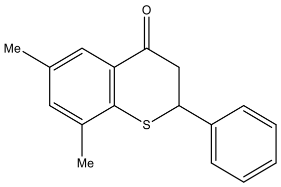
O
Me
S
Me

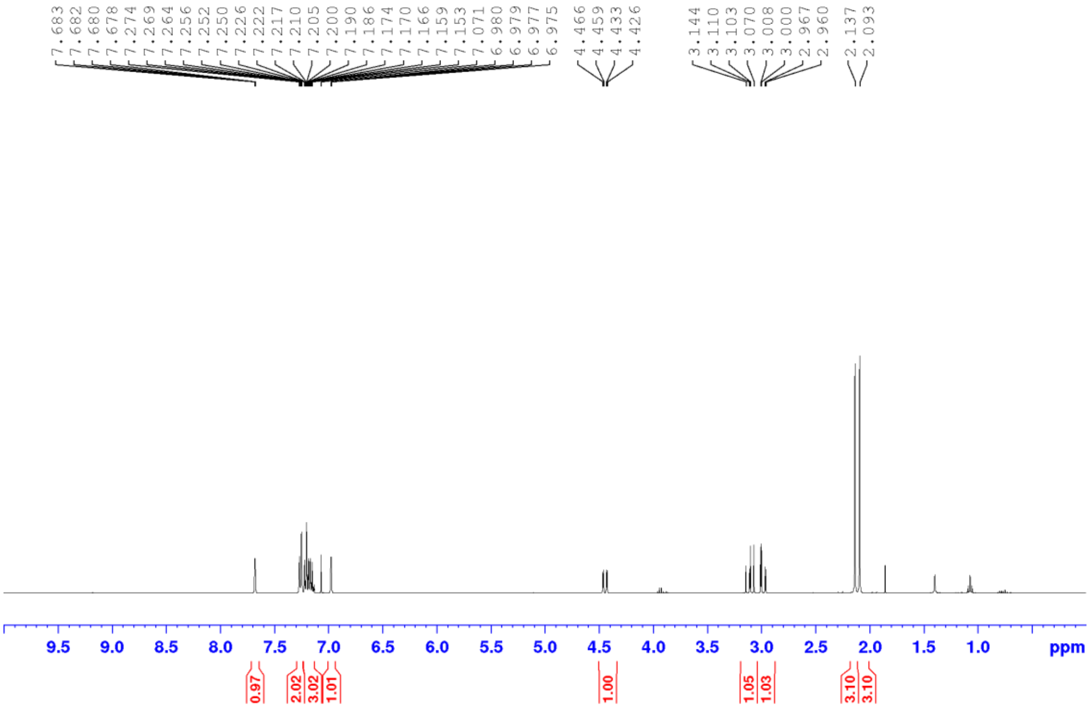
7.683
7.682
7.680
7.678
7.274
7.269
7.264
7.256
7.252
7.250
7.226
7.222
7.217
7.210
7.205
7.200
7.190
7.186
7.174
7.170
7.166
7.159
7.153
7.071
6.980
6.979
6.977
6.975
4.466
4.459
4.433
4.426
3.144
3.110
3.103
3.070
3.008
3.000
2.967
2.960
2.137
2.093
9.5 9.0 8.5 8.0 7.5 7.0 6.5 6.0 5.5 5.0 4.5 4.0 3.5 3.0 2.5 2.0 1.5 1.0 ppm
0.97
2.02
3.02
1.01
1.00
1.05
1.03
3.10
3.10

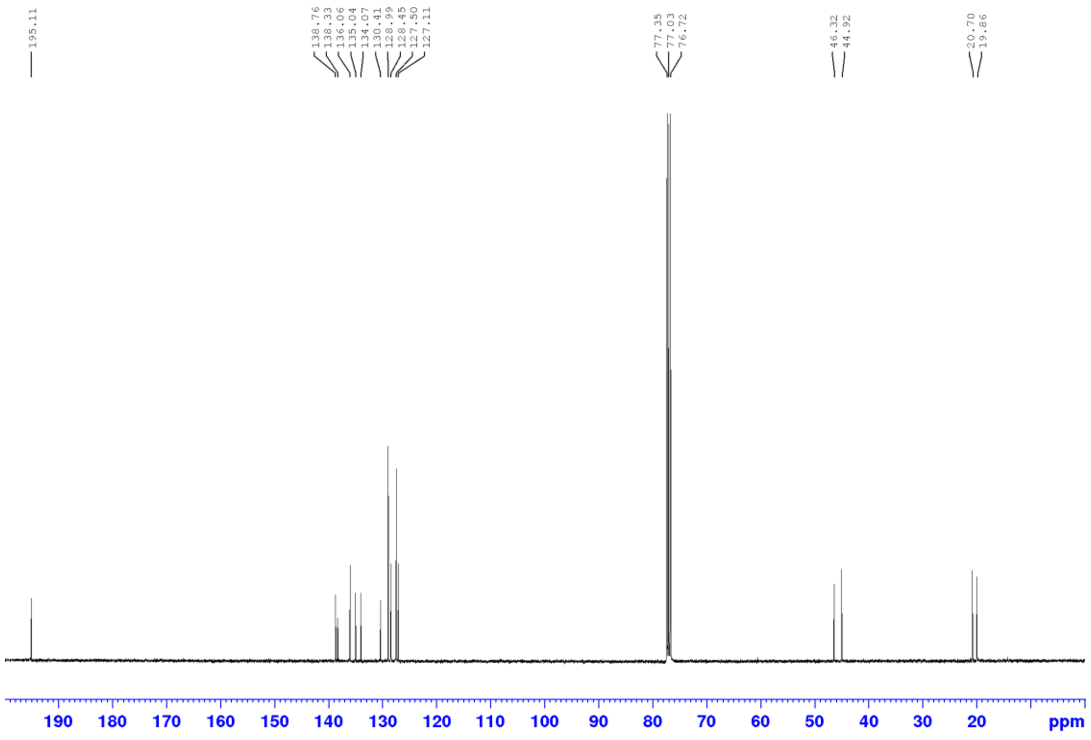
195.11
138.76
138.33
136.06
135.04
134.07
130.41
128.99
128.45
127.50
127.11
77.35
77.03
76.72
46.32
44.92
20.70
19.86
190 180 170 160 150 140 130 120 110 100 90 80 70 60 50 40 30 20 ppm

3Ka

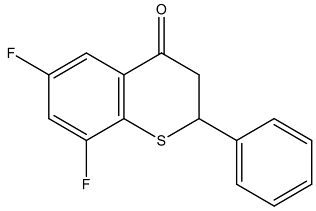
O
F
S
F

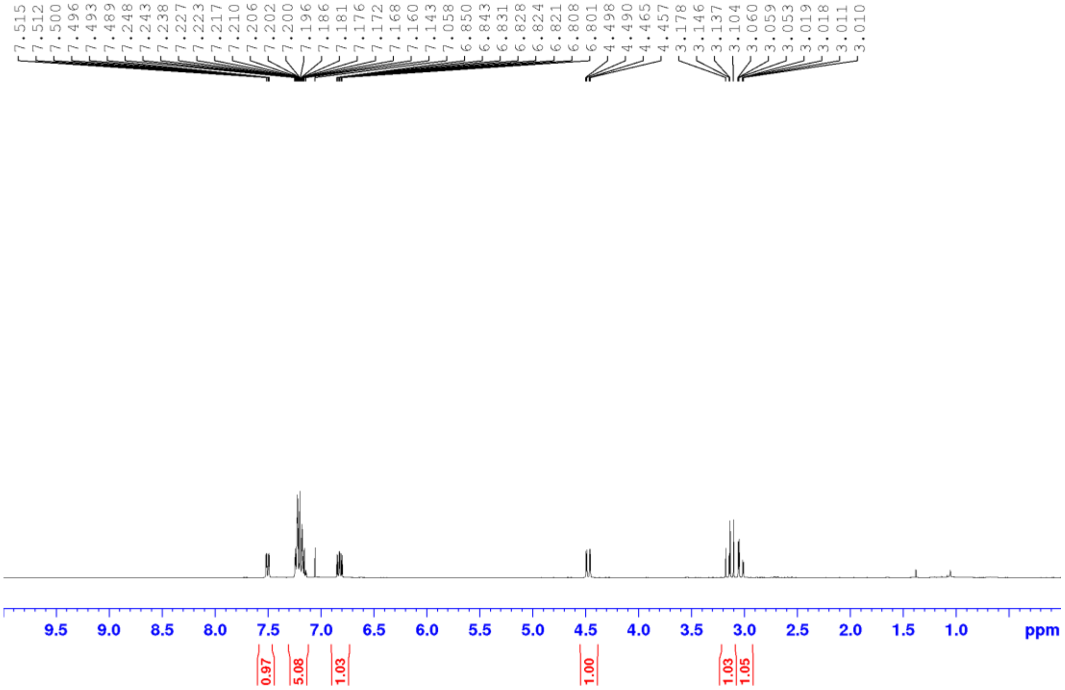
7.515
7.512
7.500
7.496
7.493
7.489
7.248
7.243
7.238
7.227
7.223
7.217
7.210
7.206
7.202
7.200
7.196
7.186
7.181
7.176
7.172
7.168
7.160
7.143
7.058
6.850
6.843
6.831
6.828
6.824
6.821
6.808
6.801
4.498
4.490
4.465
4.457
3.178
3.146
3.137
3.104
3.060
3.059
3.053
3.019
3.018
3.011
3.010
9.5 9.0 8.5 8.0 7.5 7.0 6.5 6.0 5.5 5.0 4.5 4.0 3.5 3.0 2.5 2.0 1.5 1.0 ppm
0.97
5.08
1.03
1.00
1.03
1.05

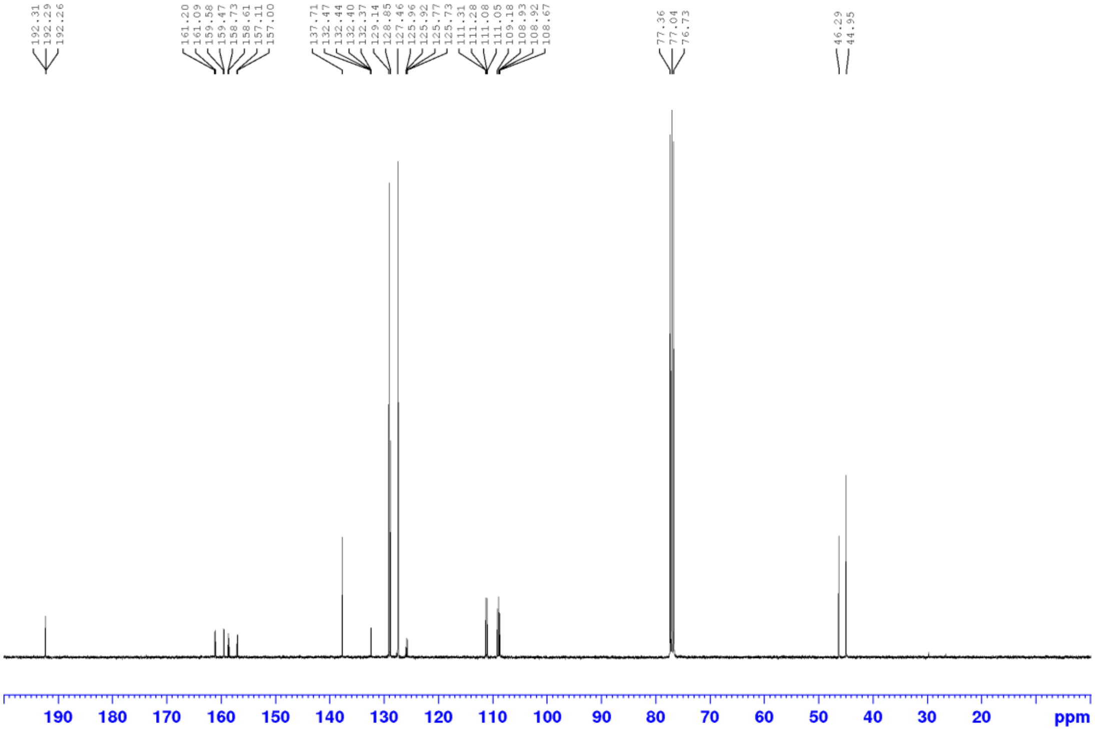
192.31
192.29
192.26
161.20
161.09
159.58
159.47
158.73
158.61
157.11
157.00
137.71
132.47
132.44
132.40
132.37
129.14
128.85
127.46
125.96
125.92
125.77
125.73
111.31
111.28
111.08
111.05
109.18
108.93
108.92
108.67
77.36
77.04
76.73
46.29
44.95
190 180 170 160 150 140 130 120 110 100 90 80 70 60 50 40 30 20 ppm

3Ka

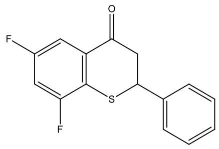
O
F
S
F

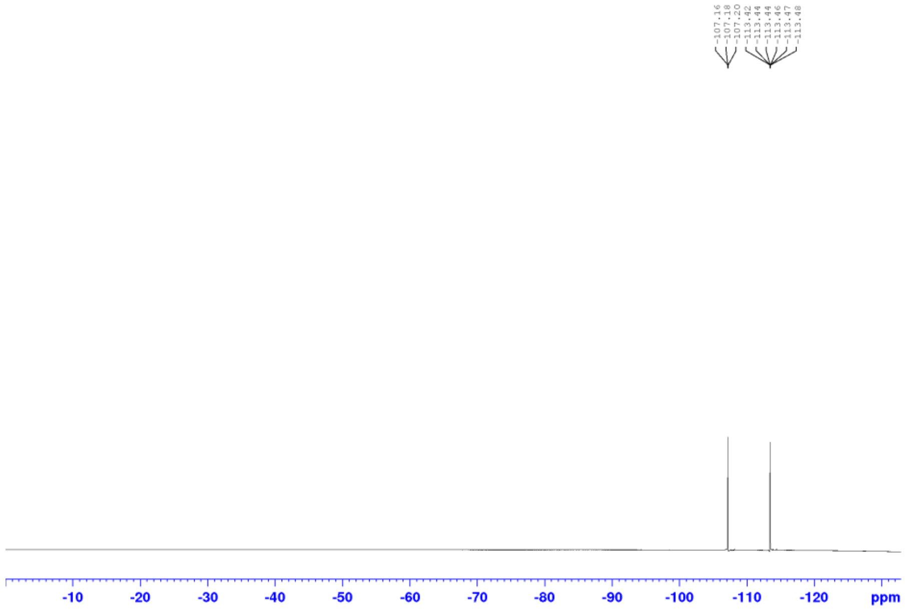
-107.16
-107.18
-107.20
-113.42
-113.44
-113.44
-113.46
-113.47
-113.48
-10
-20
-30
-40
-50
-60
-70
-80
-90
-100
-110
-120
ppm

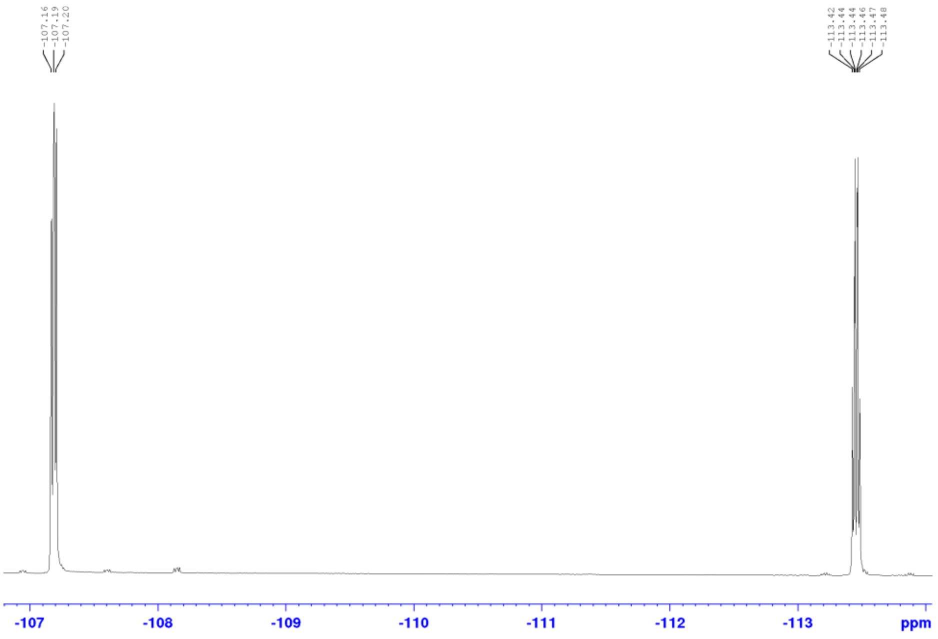
-107.16
-107.19
-107.20
-113.42
-113.44
-113.44
-113.46
-113.47
-113.48
-107
-108
-109
-110
-111
-112
-113
ppm

3Ka

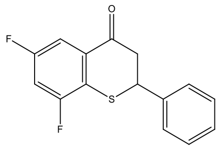
O
F
F
S

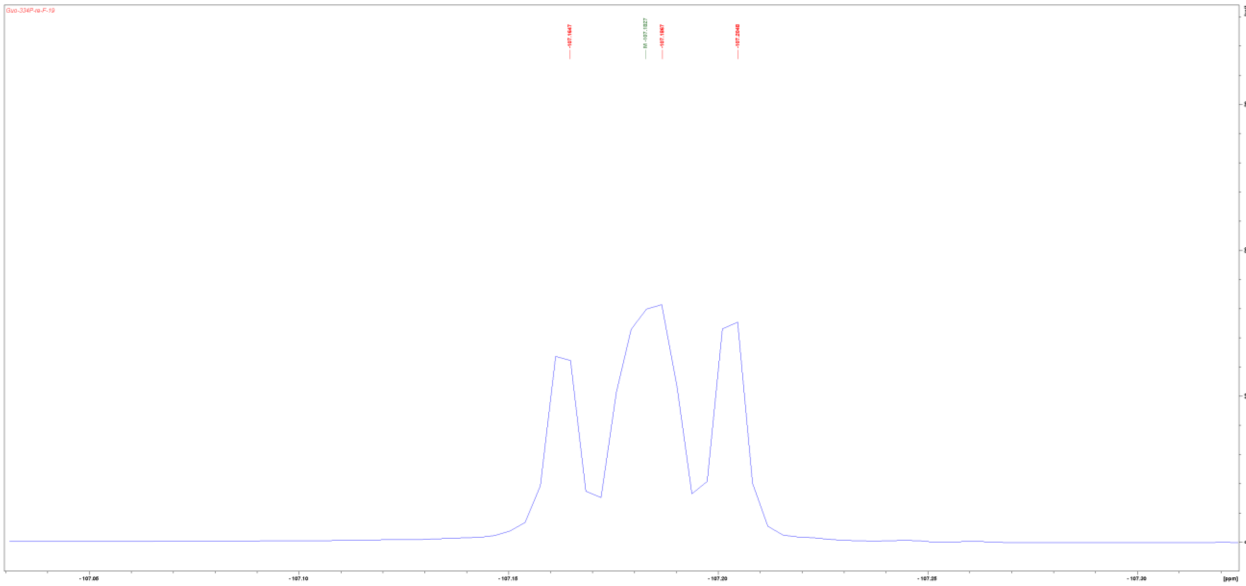

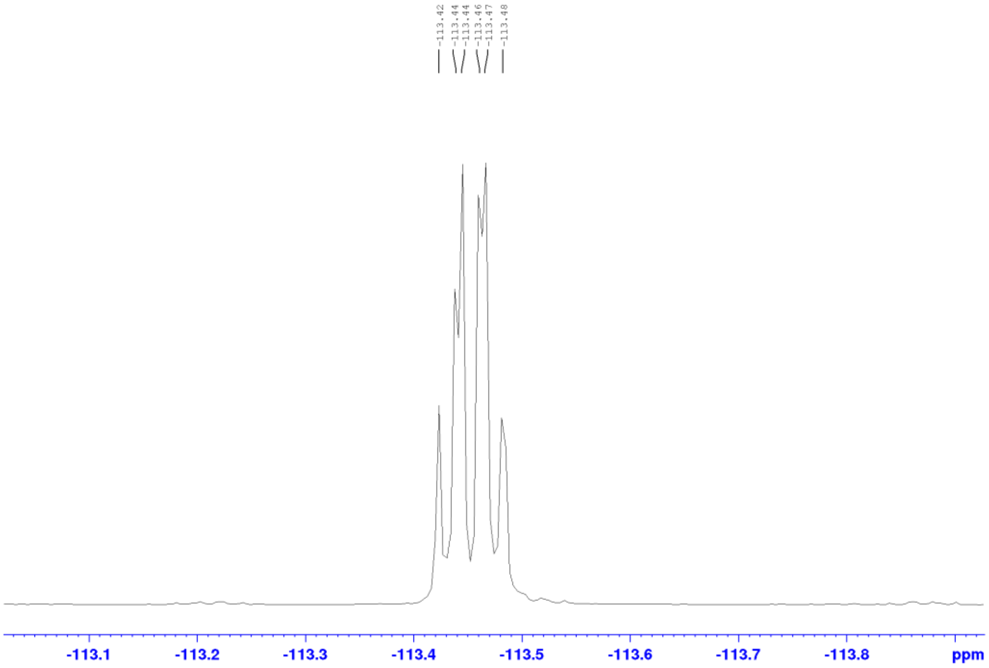
-113.42
-113.44
-113.44
-113.46
-113.47
-113.48
-113.1
-113.2
-113.3
-113.4
-113.5
-113.6
-113.7
-113.8
ppm

3La

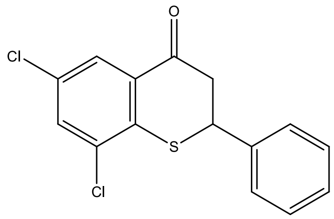
O
Cl
S
Cl

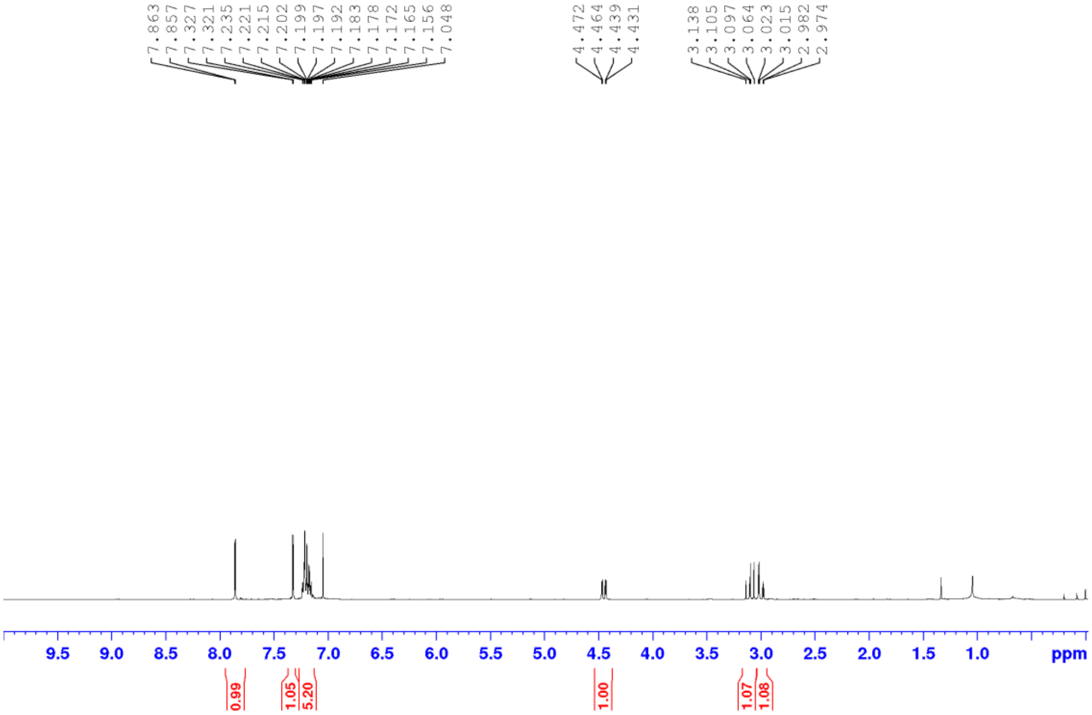
7.863
7.857
7.327
7.321
7.235
7.221
7.215
7.202
7.199
7.197
7.192
7.183
7.178
7.172
7.165
7.156
7.048
4.472
4.464
4.439
4.431
3.138
3.105
3.097
3.064
3.023
3.015
2.982
2.974
9.5
9.0
8.5
8.0
7.5
7.0
6.5
6.0
5.5
5.0
4.5
4.0
3.5
3.0
2.5
2.0
1.5
1.0
ppm
0.99
1.05
5.20
1.00
1.07
1.08

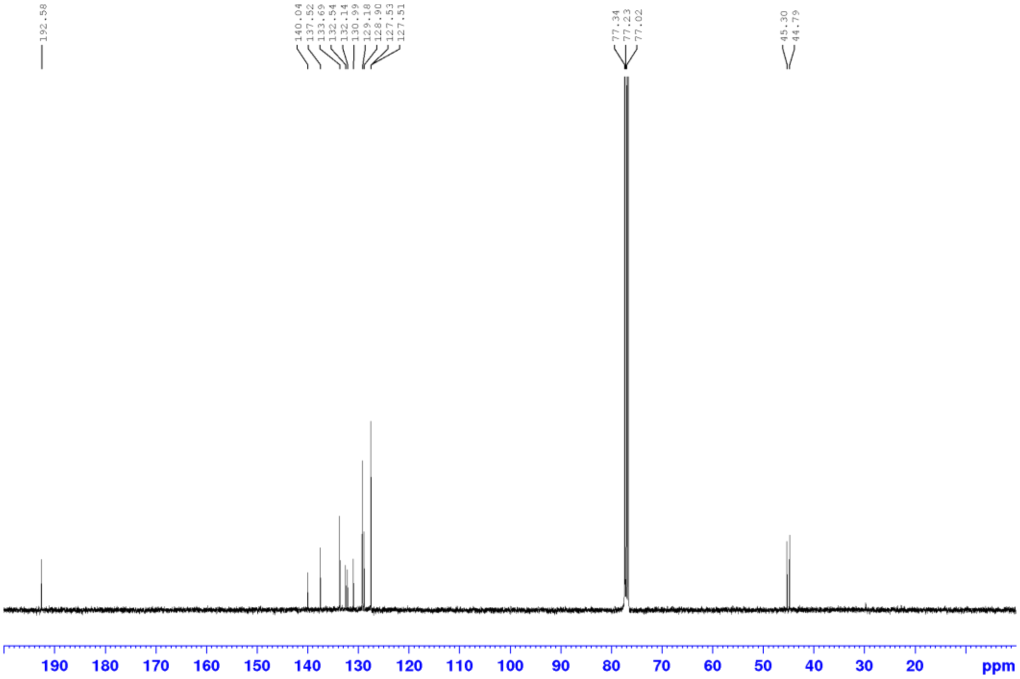
192.58
140.04
137.52
133.69
132.54
132.14
130.99
129.18
128.90
127.53
127.51
77.34
77.23
77.02
45.30
44.79
190
180
170
160
150
140
130
120
110
100
90
80
70
60
50
40
30
20
ppm

3Ma

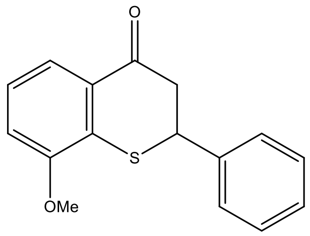

O
S
OMe

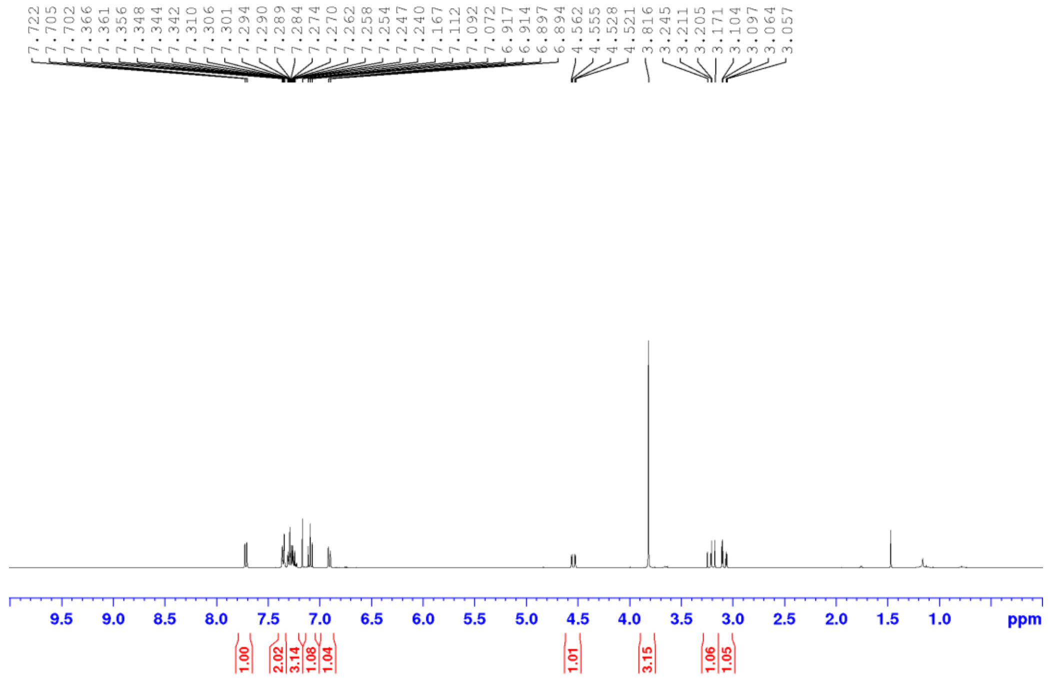

7.722 7.705 7.702 7.366 7.361 7.356 7.348 7.344 7.342 7.310 7.306 7.301 7.294 7.290 7.289 7.284 7.274 7.270 7.262 7.258 7.254 7.247 7.240 7.167 7.112 7.092 7.072 6.917 6.914 6.897 6.894 4.562 4.555 4.528 4.521 3.816 3.245 3.211 3.205 3.171 3.104 3.097 3.064 3.057
9.5 9.0 8.5 8.0 7.5 7.0 6.5 6.0 5.5 5.0 4.5 4.0 3.5 3.0 2.5 2.0 1.5 1.0 ppm
1.00 2.02 3.14 1.08 1.04 1.01 3.15 1.06 1.05

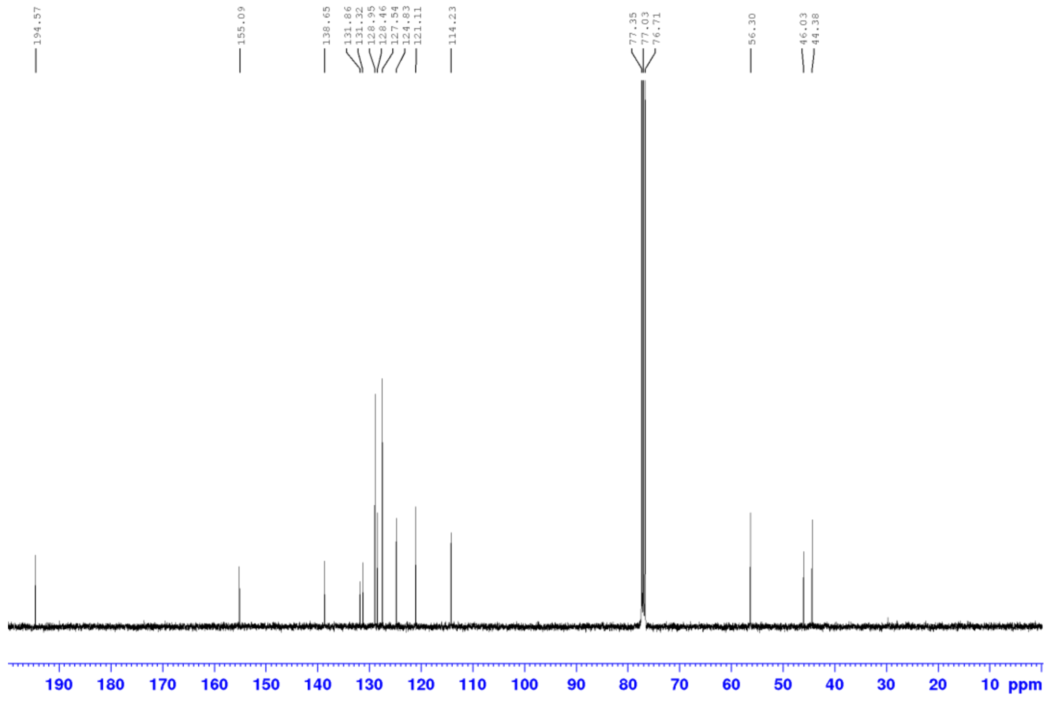

194.57 155.09 138.65 131.86 131.32 128.95 128.46 127.54 124.83 121.11 114.23 77.35 77.03 76.71 56.30 46.03 44.38
190 180 170 160 150 140 130 120 110 100 90 80 70 60 50 40 30 20 10 ppm

3Pa

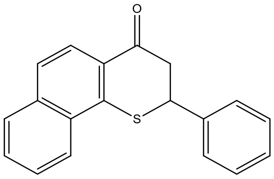

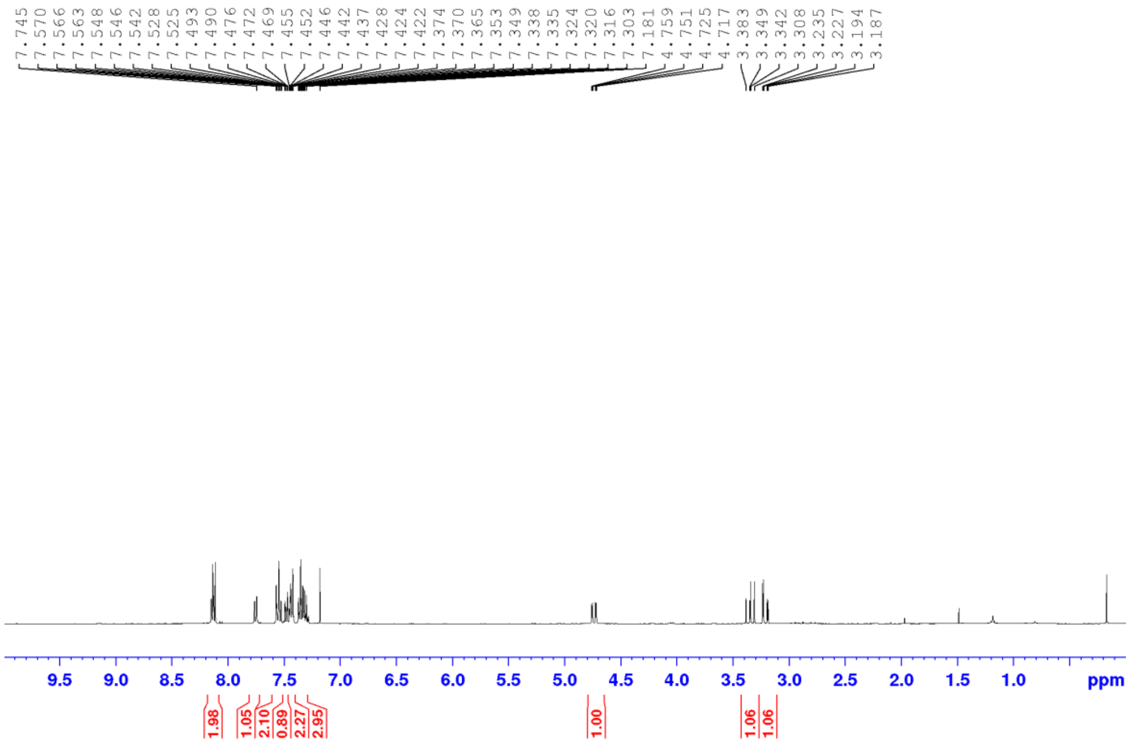

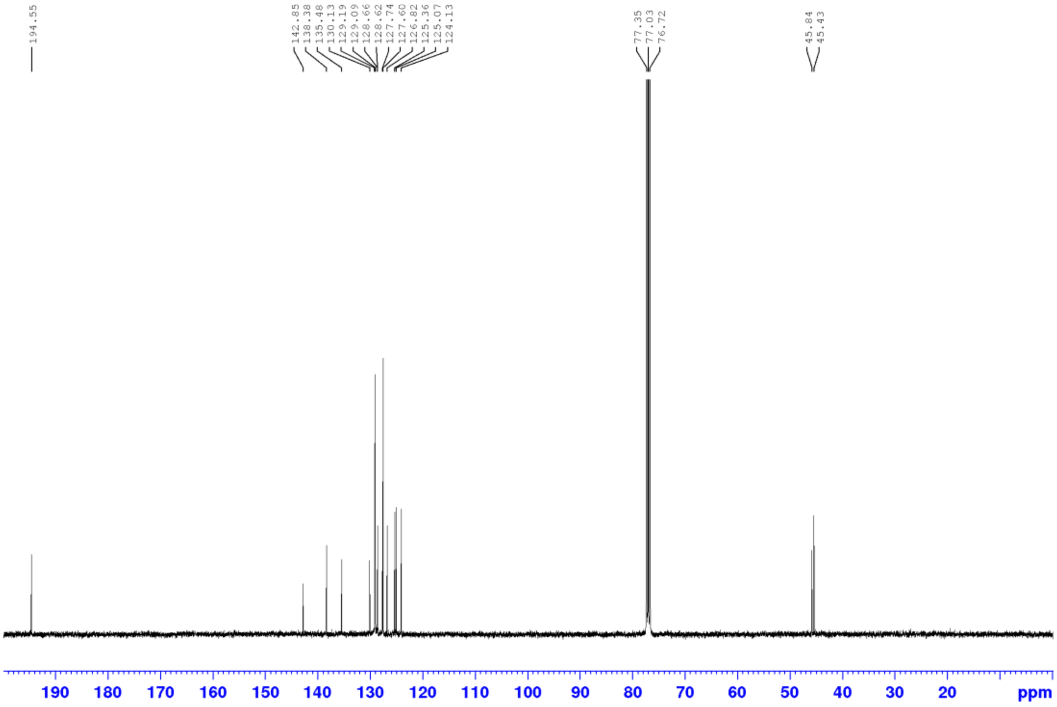

4Af

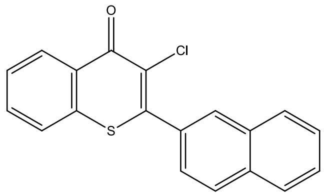
O
Cl
S

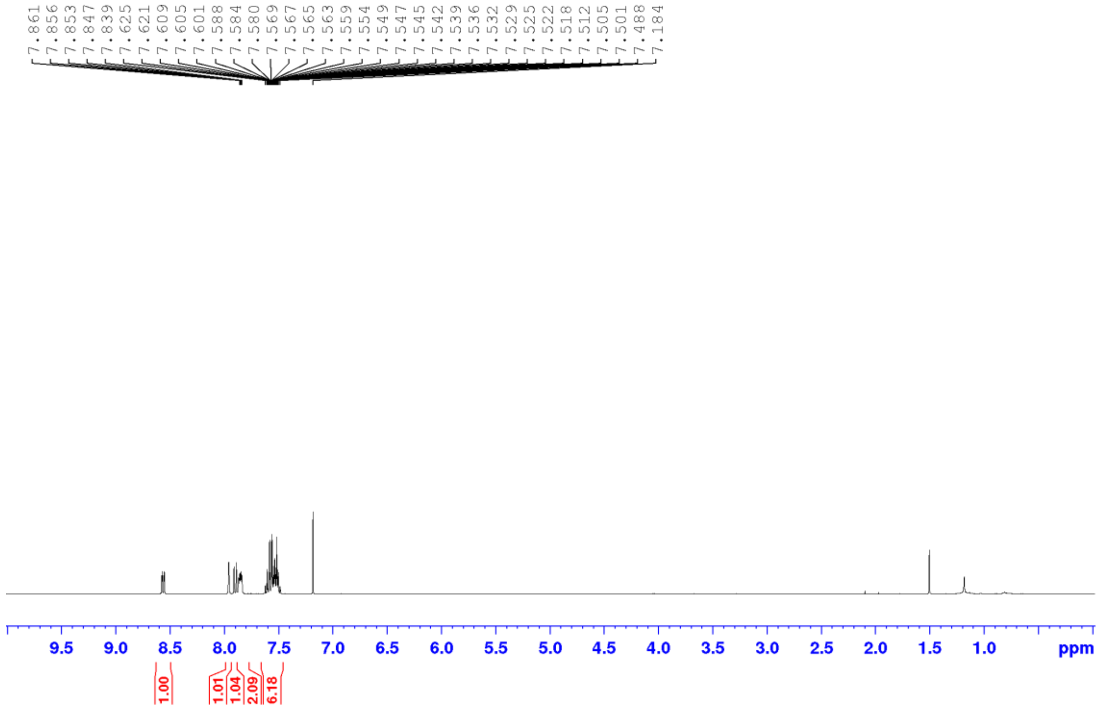
7.861 7.856 7.853 7.847 7.839 7.625 7.621 7.609 7.605 7.601 7.588 7.584 7.580 7.569 7.567 7.565 7.563 7.559 7.554 7.549 7.547 7.545 7.542 7.539 7.536 7.532 7.529 7.525 7.522 7.518 7.512 7.505 7.501 7.488 7.184
9.5 9.0 8.5 8.0 7.5 7.0 6.5 6.0 5.5 5.0 4.5 4.0 3.5 3.0 2.5 2.0 1.5 1.0 ppm
1.00 1.01 1.04 2.09 6.18

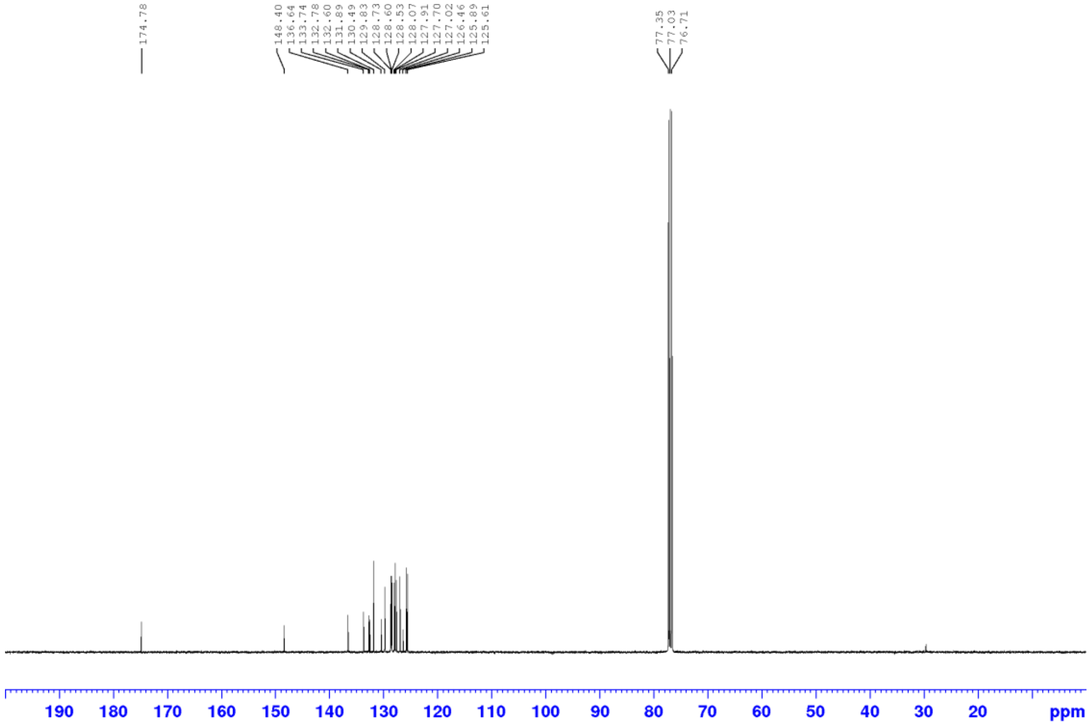
174.78
148.40 136.64 133.74 132.78 132.60 131.89 130.49 129.83 128.73 128.60 128.53 128.07 127.91 127.70 127.02 126.46 125.89 125.61
77.35 77.03 76.71
190 180 170 160 150 140 130 120 110 100 90 80 70 60 50 40 30 20 ppm

5Ac

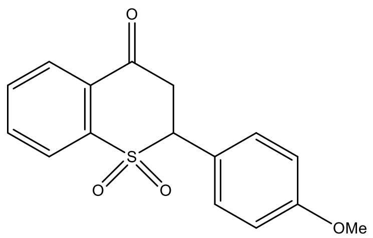
O
S
O
O
OMe

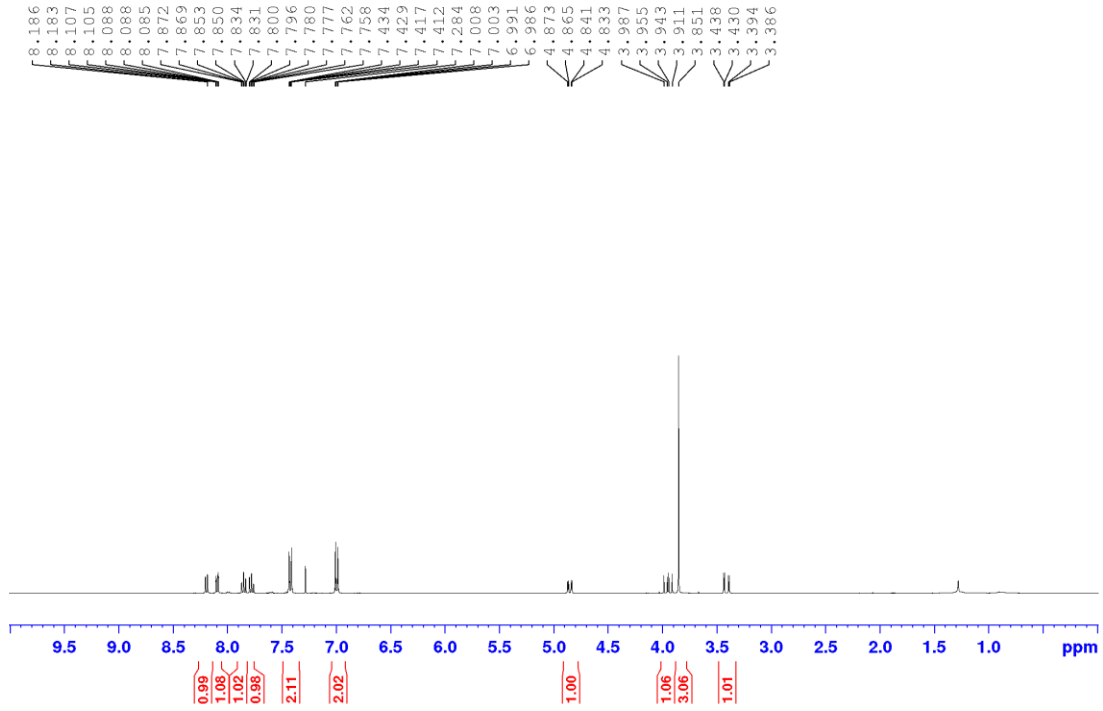
8.186
8.183
8.107
8.105
8.088
8.088
8.085
7.872
7.869
7.853
7.850
7.834
7.831
7.800
7.796
7.780
7.777
7.762
7.758
7.434
7.429
7.417
7.412
7.284
7.008
7.003
6.991
6.986
4.873
4.865
4.841
4.833
3.987
3.955
3.943
3.911
3.851
3.438
3.430
3.394
3.386
9.5 9.0 8.5 8.0 7.5 7.0 6.5 6.0 5.5 5.0 4.5 4.0 3.5 3.0 2.5 2.0 1.5 1.0 ppm
0.99
1.08
1.02
0.98
2.11
2.02
1.00
1.06
3.06
1.01

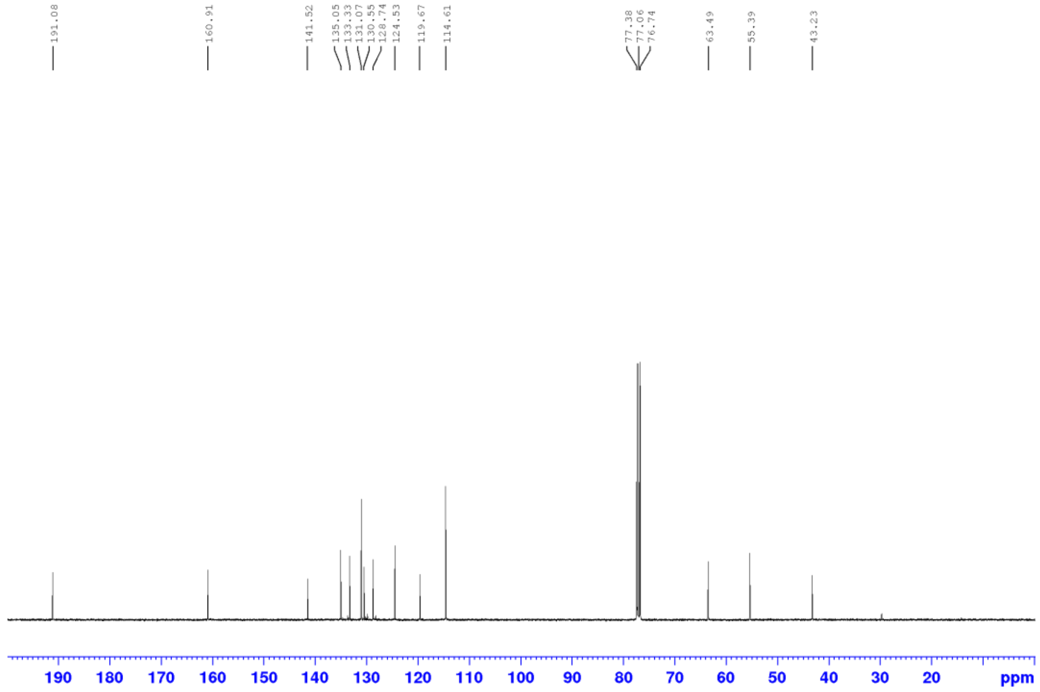
191.08
160.91
141.52
135.05
133.33
131.07
130.55
128.74
124.53
119.67
114.61
77.38
77.06
76.74
63.49
55.39
43.23
190 180 170 160 150 140 130 120 110 100 90 80 70 60 50 40 30 20 ppm

5Ae

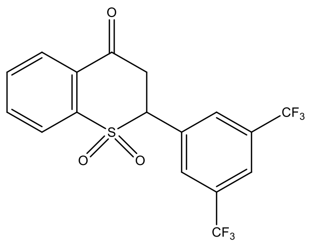
O
CF_3
S
O
O
CF_3

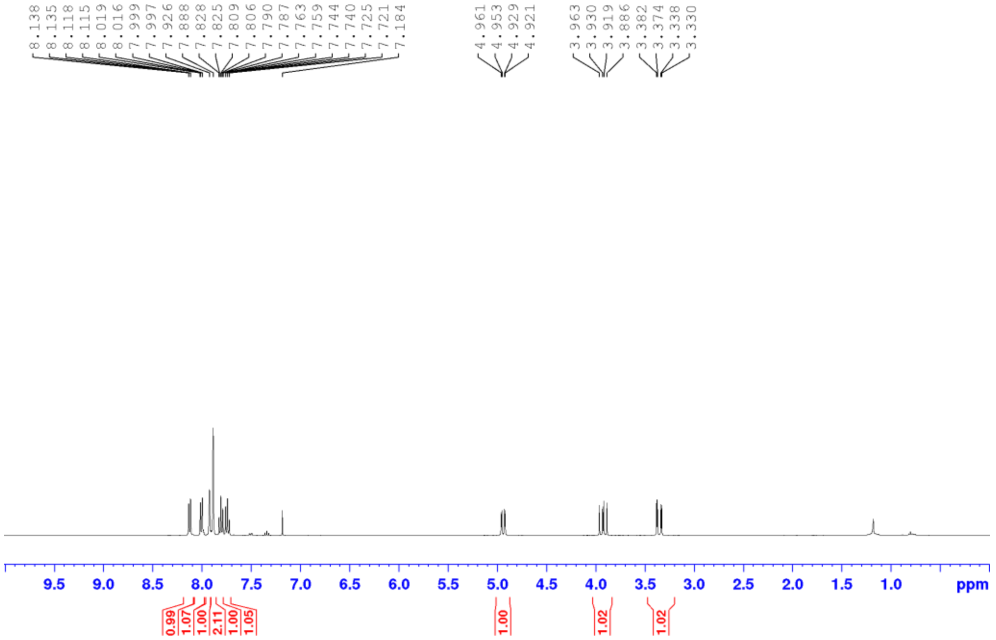
8.138
8.135
8.118
8.115
8.019
8.016
7.999
7.997
7.926
7.888
7.828
7.825
7.809
7.806
7.790
7.787
7.763
7.759
7.744
7.740
7.725
7.721
7.184
4.961
4.953
4.929
4.921
3.963
3.930
3.919
3.886
3.382
3.374
3.338
3.330
9.5 9.0 8.5 8.0 7.5 7.0 6.5 6.0 5.5 5.0 4.5 4.0 3.5 3.0 2.5 2.0 1.5 1.0 ppm
0.99
1.07
1.00
2.11
1.00
1.05
1.00
1.02
1.02

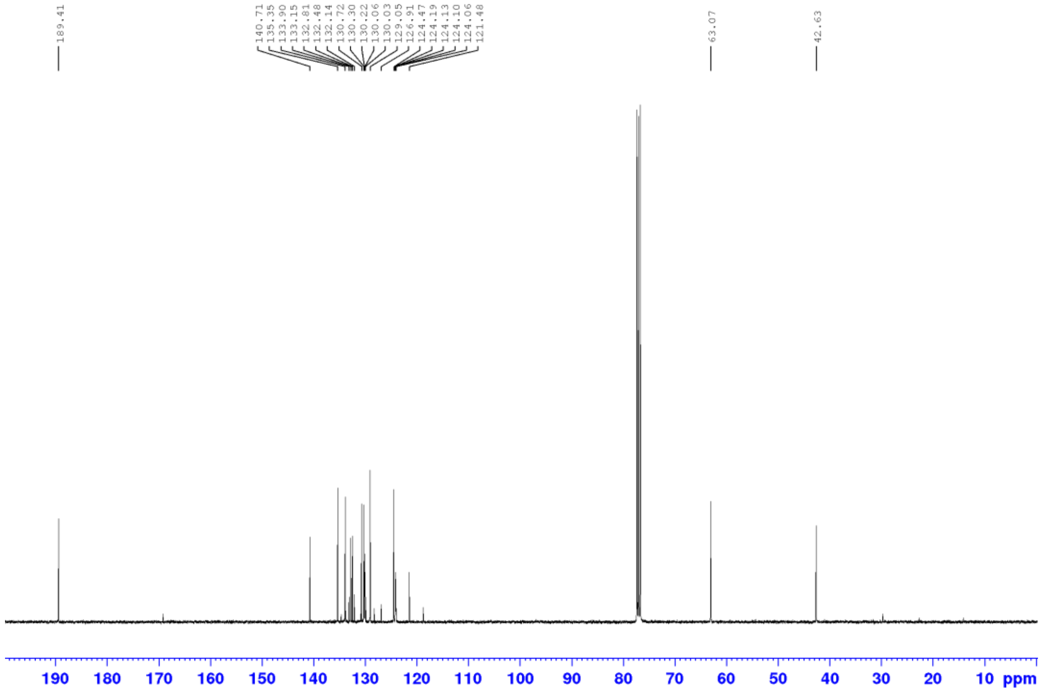
189.41
140.71
135.35
133.90
133.15
132.81
132.48
132.14
130.72
130.30
130.22
130.06
130.03
129.05
126.91
124.47
124.19
124.13
124.10
124.06
121.48
63.07
42.63
190 180 170 160 150 140 130 120 110 100 90 80 70 60 50 40 30 20 10 ppm

5Ae

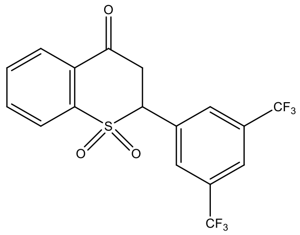

O
S
O
O
CF3
CF3

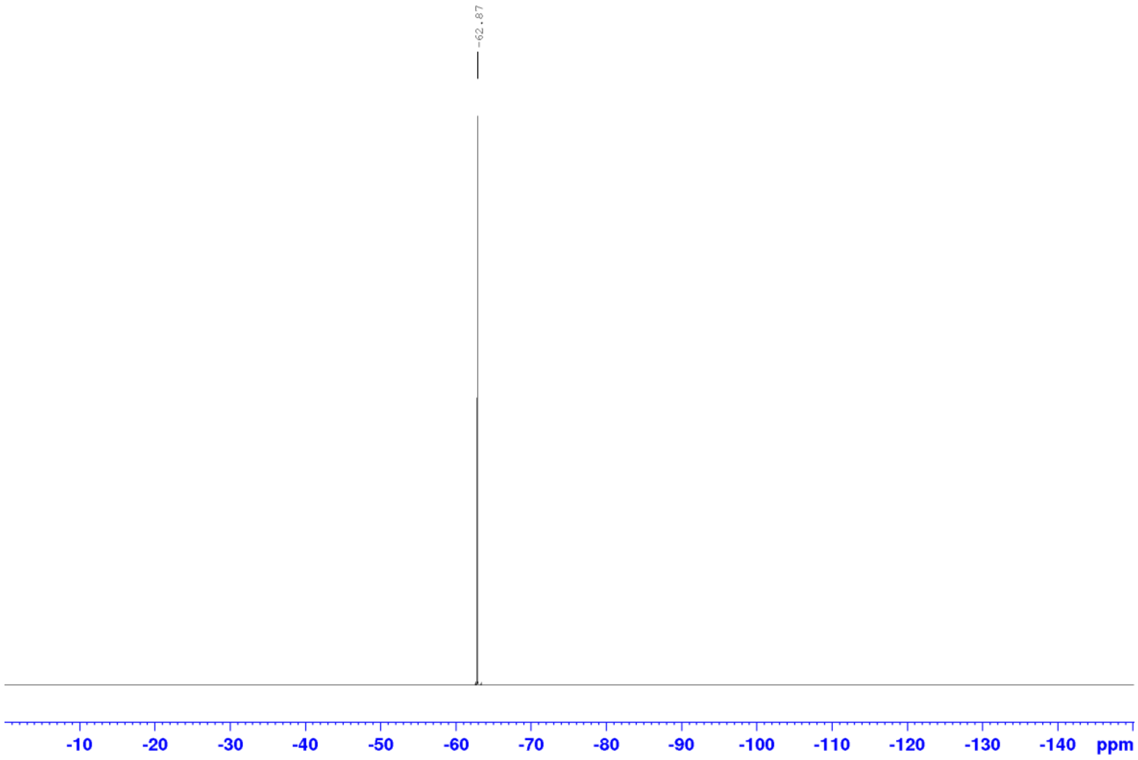

-62.87
-10
-20
-30
-40
-50
-60
-70
-80
-90
-100
-110
-120
-130
-140
ppm

7Ab

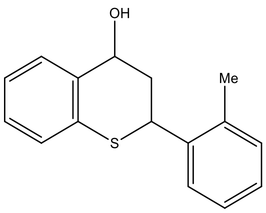

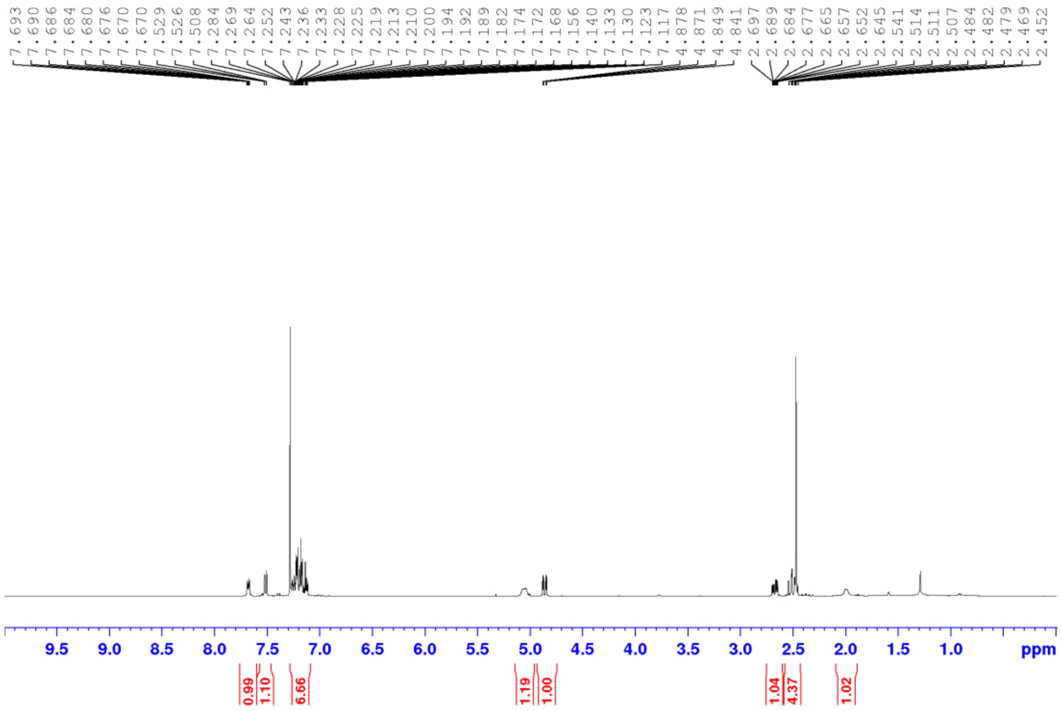

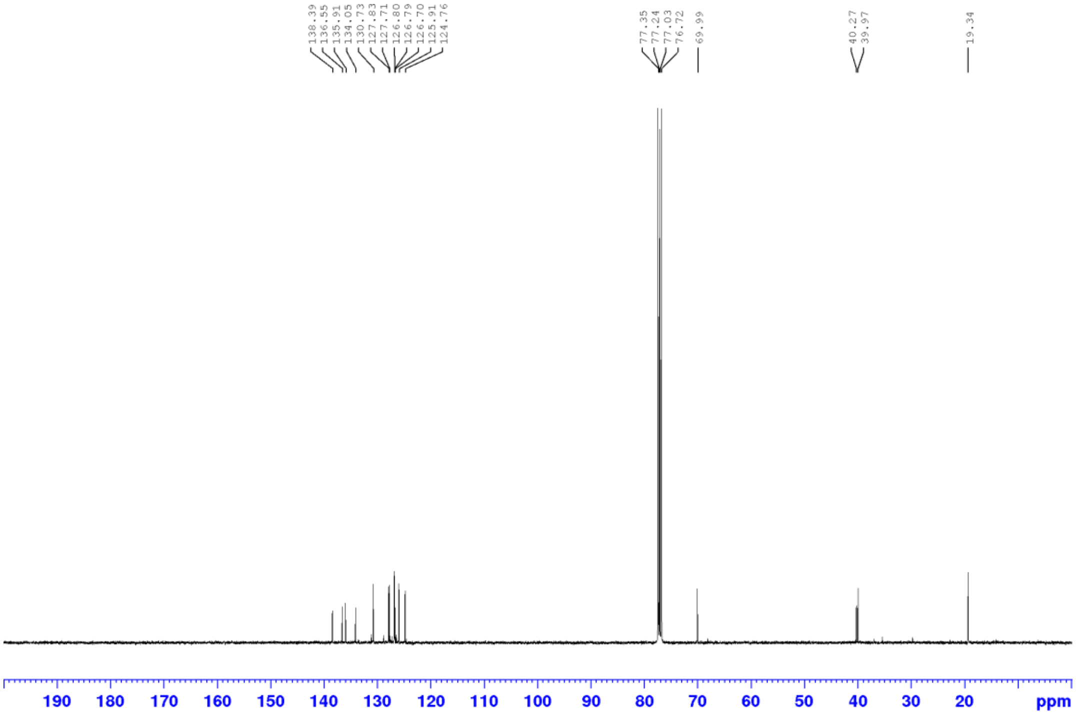

Supplement: Supplementary file 1 [file molecules-25-02128-s001.pdf]
